# Machine-Learning-Based Single-Molecule Quantification of Circulating MicroRNA Mixtures

**DOI:** 10.1021/acssensors.3c01234

**Published:** 2023-10-04

**Authors:** Jonathan Jeffet, Sayan Mondal, Amit Federbush, Nadav Tenenboim, Miriam Neaman, Jasline Deek, Yuval Ebenstein, Yohai Bar-Sinai

**Affiliations:** † School of Physics and Astronomy, Raymond and Beverly Sackler Faculty of Exact Sciences, Tel Aviv University, Tel Aviv 6997801, Israel; ‡ School of Chemistry, Raymond and Beverly Sackler Faculty of Exact Sciences, Tel Aviv University, Tel Aviv 6997801, Israel; § Department of Biomedical Engineering, Fleischman Faculty of Engineering, Tel Aviv University, Tel Aviv 6997801, Israel; ∥ Center for Nanoscience and Nanotechnology, Tel Aviv University, Tel Aviv 6997801, Israel; ⊥ The Center for Physics and Chemistry of Living Systems, Tel Aviv University, Tel Aviv 6997801, Israel; # Department of Hematology, Tel Aviv Sourasky Medical Center, Tel Aviv 6423906, Israel; ¶ Center for AI & Data Science (TAD), Tel Aviv University, Tel Aviv 6997801, Israel

**Keywords:** spectral imaging, machine learning, circulating microRNA, single-molecule, cancer diagnostics

## Abstract

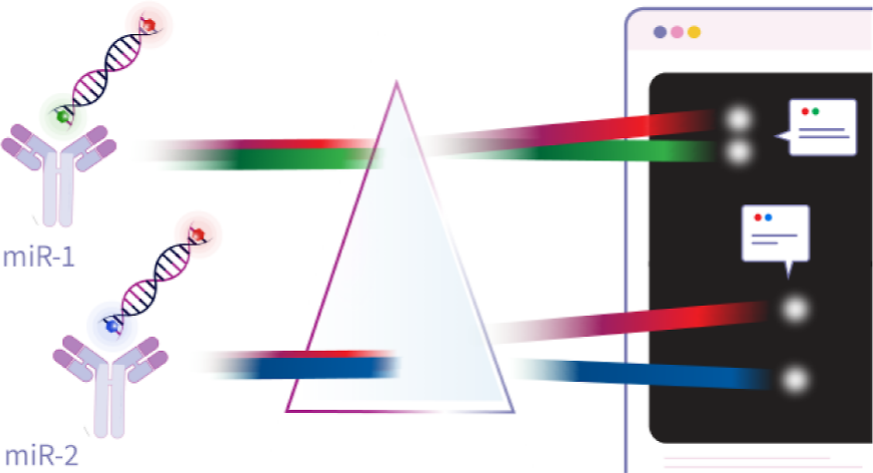

MicroRNAs (miRs) are small noncoding RNAs that regulate gene expression and are emerging as powerful indicators of diseases. MiRs are secreted in blood plasma and thus may report on systemic aberrations at an early stage via liquid biopsy analysis. We present a method for multiplexed single-molecule detection and quantification of a selected panel of miRs. The proposed assay does not depend on sequencing, requires less than 1 mL of blood, and provides fast results by direct analysis of native, unamplified miRs. This is enabled by a novel combination of compact spectral imaging and a machine learning-based detection scheme that allows simultaneous multiplexed classification of multiple miR targets per sample. The proposed end-to-end pipeline is extremely time efficient and cost-effective. We benchmark our method with synthetic mixtures of three target miRs, showcasing the ability to quantify and distinguish subtle ratio changes between miR targets.

MicroRNAs (miRs) are evolutionarily conserved, 18–25 nucleotide-long noncoding RNAs that regulate the translation of mRNA.^1,2^ MiRs regulate the transcription of up to 60% of all human protein-coding genes and are therefore crucial for cellular function.^3^ Aberrant miR levels reflect the physiological state of cancer cells and correlate closely with tumor origin and stage.^4^ Importantly, miRs are highly present in circulation, are protected from RNases digestion by extracellular vesicles or protein-binding, and are tightly related to oncogenesis, making them promising candidates for biomarkers.^5−9^ Expression analyses of miRs circulating in blood emerge as promising and complementing clinical tools for early molecular diagnostics and follow-up.^10^ Relative expression-level signatures of small panels consisting of <10 targets of carefully selected miRs have significant diagnostic and prognostic power.^7,11,12^ Such disease-specific panels are already well established in the literature^7,13^ and dedicated databases.^14−16^

However, current mainstream methodologies for quantifying miR expression are neither optimized nor designed for the simultaneous quantification of several miR targets.^17−20^ Standard methodologies for miR expression quantification, such as quantitative reverse transcriptase polymerase chain reaction (qRT-PCR), RNA sequencing (RNAseq), and microarrays,^20^ rely on PCR amplification for analysis. Due to their short sequence length, miRs are not easily amplified by PCR, introducing bias into such expression analysis.^21,22^ Each of these methods has its drawbacks when quantifying small panels of miRs: qRT-PCR has limited multiplexing capabilities such that analyzing the expression of more than three miR targets often requires extensive optimizations and validations and is limited in sample throughput.^23^ Standard RNAseq requires nonspecific sequencing of all small RNAs and therefore suffers from long turnaround times and relatively high costs per target when small panels of targets are needed. Furthermore, due to the large abundance variation between miR targets in body fluids,^24^ deep sequencing is required for expression profiling of rare miRs. Microarrays are good for multiplexing a large number of targets at relatively low costs but suffer from low sensitivity and specificity and are difficult to use for absolute quantification.^19,25−27^

Native miR detection mitigates these biases and is currently possible with the commercial NanoString system;^28^ however, this solution is more suitable for panels consisting of hundreds of miRs and is generally excessive and expensive for panels of several miRs of interest.^29^

Recent studies have demonstrated the capability to optically detect miRs at single-molecule resolutions.^30−33^ Nevertheless, their multiplexing capabilities are currently limited to two miR targets simultaneously. In clinical applications, such as diagnostics and follow-up, there is a need to evaluate miR panels consisting of multiple targets in a fast, cost-effective, and sensitive manner.

Here, we introduce our method, miR Analysis by spectral Classification LEarning (miRACLE), a method for multiplexed single-molecule detection and profiling designed for small panels of up to 11 disease-associated miRs.

The miRACLE’s pipeline combines four key components that will be presented in the following section: (i) capturing targeted miRs by complementary DNA probes labeled with a distinct fluorophore pair; (ii) specific miR targets immobilization using anti-RNA/DNA hybrid antibody^34^ and subsequent washing of excess probes and background; (iii) compact spectral imaging using CoCoS microscopy;^35^ and (iv) a dedicated image processing tool for detection and classification of all miR targets at single-molecule sensitivity.

## Results and Discussion

**Figure 1:**
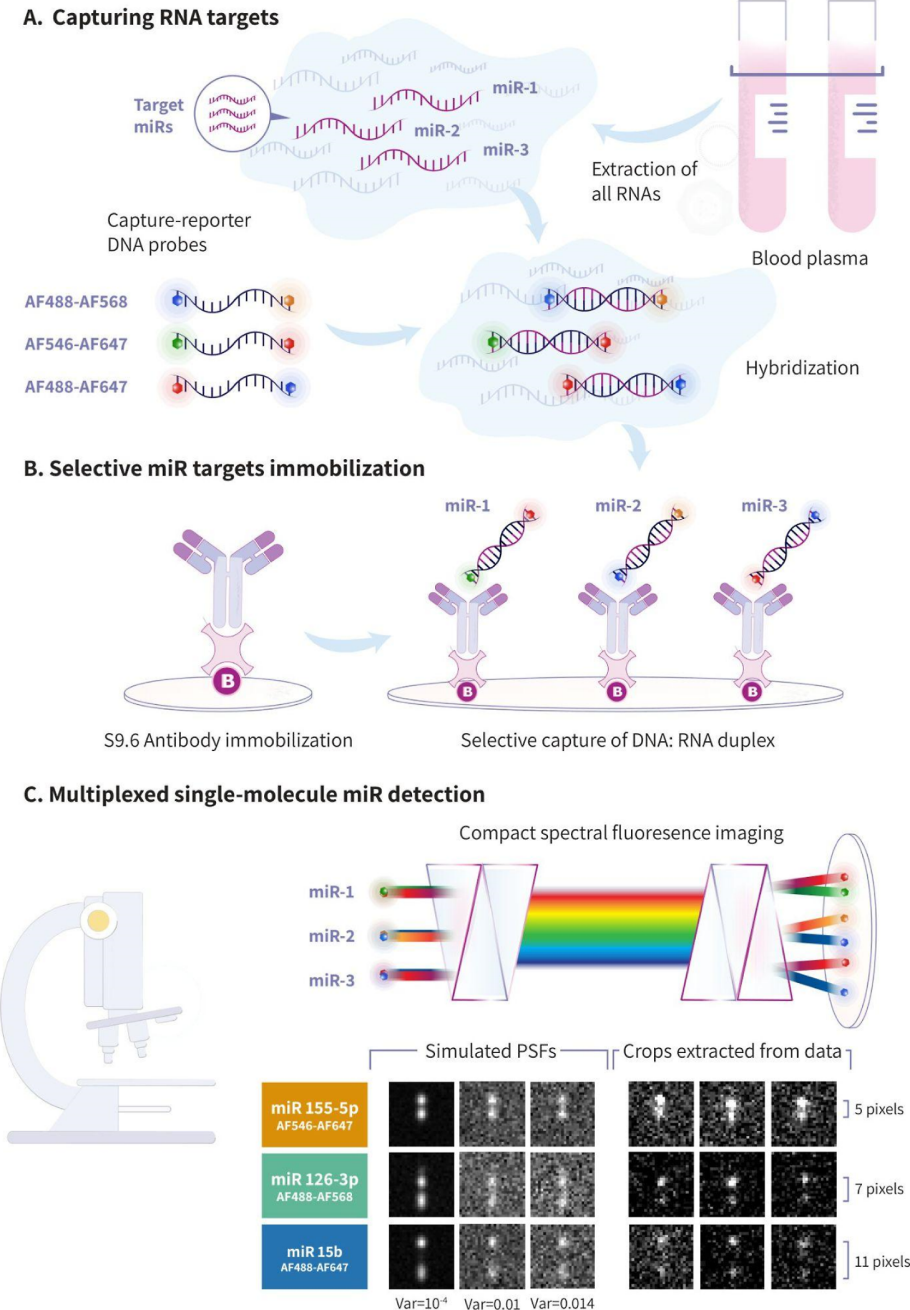
MiRACLE scheme: (A) total RNA is extracted from plasma samples, and only the selected miR targets are specifically hybridized with DNA capture-reporter probes. Each reporter has a unique fluorophore-pair combination (fluorophores names are displayed to the left, Alexa Fluor—AF). (B) Hybridized target miR: reporter DNA complexes are selectively captured on a glass surface by an Anti-DNA-RNA hybrid [S9.6] antibody, while all excess probes and molecules are washed away. The glass antibody surfaces are prepared in-house using biotin–streptavidin binding and PEG passivation. (C) Captured targets are then imaged using a compact spectral imaging microscope module (CoCoS), which allows the capture of all fluorescent probes in the FOV with a single frame (top). The miRs are classified according to the distinctive spectral signature of each reporter’s fluorophore-pair (the fluorophores’ identities are depicted at the bottom of each colored box on the left), which can be simulated (left) with varying Gaussian noise distributions (induced Gaussian noise variance is depicted at the bottom) to a high extent of resemblance with the real data (right). The expected distance between peaks is displayed to the right. All crops are presented with the same brightness and contrast settings.

The first goal in our miRACLE pipeline is to isolate and quantify only the miR targets relevant for a disease-specific diagnosis. This focused detection allows for increasing the signal-to-noise ratio (SNR) by reducing the background noise, enhancing the dynamic range of the method, and reducing the experimental costs (see Table S1). This goal is achieved in two sequential steps. First, specific miR targets are captured and tagged using DNA capture-reporter probes composed of ∼30 bp long sequences and labeled with unique combinations of two fluorophores. These probes are designed to complement specific miR targets (Figure 1A), offering target recognition specificity with single nucleotide sensitivity.^31,33^ Thus, upon hybridization with their targets, each miR has a unique spectral signature acting as a “spectral barcode” that discloses their identity. When required, the target recognition specificity can be further improved by implementing locked nucleic acids in the probe design.^30,36^

In the following step, we use selective capturing and immobilization of the DNA/RNA hybrids to isolate the targets from the nonhybridized probes and any autofluorescing molecules in the RNA extract. DNA/RNA hybrids that correspond to hybridized miR targets are specifically captured on microscope coverslips functionalized with a monoclonal Anti-DNA-RNA hybrid [S9.6] antibody,^31,34^ allowing for the selective imaging of only the target miRs (Figure 1B). After the miR-reporter constructs are captured on the surface, subsequent washes remove all excess unhybridized reporter probes, thus significantly reducing background noise. This capture process eliminates the nonspecific binding of reporter probes, as was thoroughly validated previously^31,34^ and in control experiments (see Figures S1–S4). As a result, this approach enables a sensitive readout of the relevant targets.

In order to validate the miRACLE concept, we used three synthetic miR targets at physiological concentrations^37^ and their corresponding DNA probes (sequences are provided in Supporting Information note 2). First, we hybridized the three miRs each with their complementary probes and immobilized them separately on three different surfaces. These experiments provided a data set of distinguished probes for training the imaging, detection, and classification process. Next, we hybridized the three probes in two separate mixtures containing all three miR targets. The mixed samples were mixed together at volume ratios of 1:1:1 and 2:5:3 for miR-15b-5p/miR-155-5p/miR-126-3p, respectively. These mixture experiments are used to benchmark the miRACLE pipeline.

The third step in the miRACLE process is reading out the spectral barcodes of the miR targets. To this end, the surface-immobilized miR/reporter hybrids with their unique fluorophore-pair combinations are imaged and resolved by miRACLE’s compact spectral imaging module based on the previously introduced CoCoS system.^35^ Importantly, the two fluorophores on each probe are positioned at distances much smaller than the diffraction limit (all probes are ∼30 bp in length, which are equivalent to ∼10 nm) and therefore are captured in the image at the same physical location. To distinguish between the overlapping fluorophores, we spectrally disperse them using two prisms (Figure 1C top), slightly shifting their image position according to their spectra (Figure S5) into a combined intensity distribution on the camera’s sensor (see Figure S6 for the experimental dispersion curve converting emission wavelength to pixel displacement on the camera sensor). This results in a spectral image where the combinations of fluorophore colors are converted to unique dual-spot point-spread function (PSF) with their interspot distance indicative of their color combination (Figure 1C bottom). CoCoS allows to symmetrically rotate the prisms along the optical axis of the fluorescence emission path, offering easy control over the spectral dispersion introduced to the image (see ref (35)). Therefore, the spectral resolution is optimized for each panel of reporter probes according to its fluorophore-pairs and multiplexing needs to establish the best throughput and SNR. Optimizing the spectral dispersion enables the multiplexed registration of single-molecule miRs.

Using CoCoS, all targets are imaged simultaneously in a single frame acquisition per field-of-view (FOV, ∼130 × 130 μm^2^), reducing sample acquisition time by a factor of the number of fluorophores used and eliminating cross-color photobleaching by consecutive excitations. Importantly, spectral registration allows expanding the palette of fluorophore options available for reporter tagging and also eliminates cross-talk between color channels and the need for color channel alignment and registration. In miRACLE, each FOV contains hundreds of miRs, each detected as a unique two-spot diffraction-limited PSF corresponding to the spectral emission signature of its fluorophore-pair (Figure 1C). The unique interspot distances and their intensity distribution enable the classification of spectral PSFs and consequently facilitate visual differentiation between the target miRs. To guarantee miRACLE’s ability to multiplex and classify different miR targets, we designed a simple PSF simulator in Matlab (Figure 1C bottom). The simulator input is composed of the fluorophores’ and filter’s spectra together with the induced spectral dispersion by CoCoS (Figures S5 and S6). It then simulates the spectral PSFs with an option of adding Gaussian and Poisson noises, which provides a more realistic assessment of our probe classification capability.

The PSF simulator allowed us to input various dual-fluorophore combinations and examine their induced spectral PSF on our system. This tool enabled us to visually inspect the outcome of different fluorophore combinations (Figure S7) and assess the number of spots and distances in their induced spectral PSFs’ (selection of fluorophores, which emit in multiple spectral windows of our multiband filter, can create three and four spots intensity distributions, as shown in Figure S8). The main contribution of the simulator to miRACLE’s pipeline is the potential discovery of distinguishable fluorophore-pair combinations for the maximal multiplexing capability. Choosing fluorophore-pairs that give unique distances between PSFs’ spots and visually distinguishable intensity distributions allows one to maximize the number of probes that can be simultaneously classified in a single sample. With a crude visual inspection of the simulator results of hundreds of fluorophore combinations (Figure S7), we were able to assemble 11 fluorophore-pairs combinations that have distinguishable PSFs and interspot distances. These fluorophore-pairs could potentially allow simultaneous classification of up to 11 miR targets in a single snapshot, with the same spectral resolution and with realistic SNRs (Figure S8 and Table S2). On the other hand, the PSF simulator also allows one to find the optimal experimental setting for a specific experiment with a set probe panel, optimizing the inherent trade-off between spectral resolution, SNR, and the maximal miR density. By visually inspecting the output PSFs, the users can find the optimal spectral resolution and fluorophore-pair combinations according to the experimentally required number of miR targets.

As shown in Figure 1C, the PSF simulator results closely resemble the experimental PSF extracted from three different experiments, each imaging a single miR target at a time. The simulated and experimentally calculated interspot distances are in exact agreement, although the intensity distribution between spots differs slightly due to axial focal point chromatic aberrations that were unaccounted for in the simulations.

After image acquisition, the miRACLE pipeline proceeds with automatic analysis of the entire image data set with the aim of detecting, identifying, and quantifying the single-molecule distributions of miR targets.

**Figure 2:**
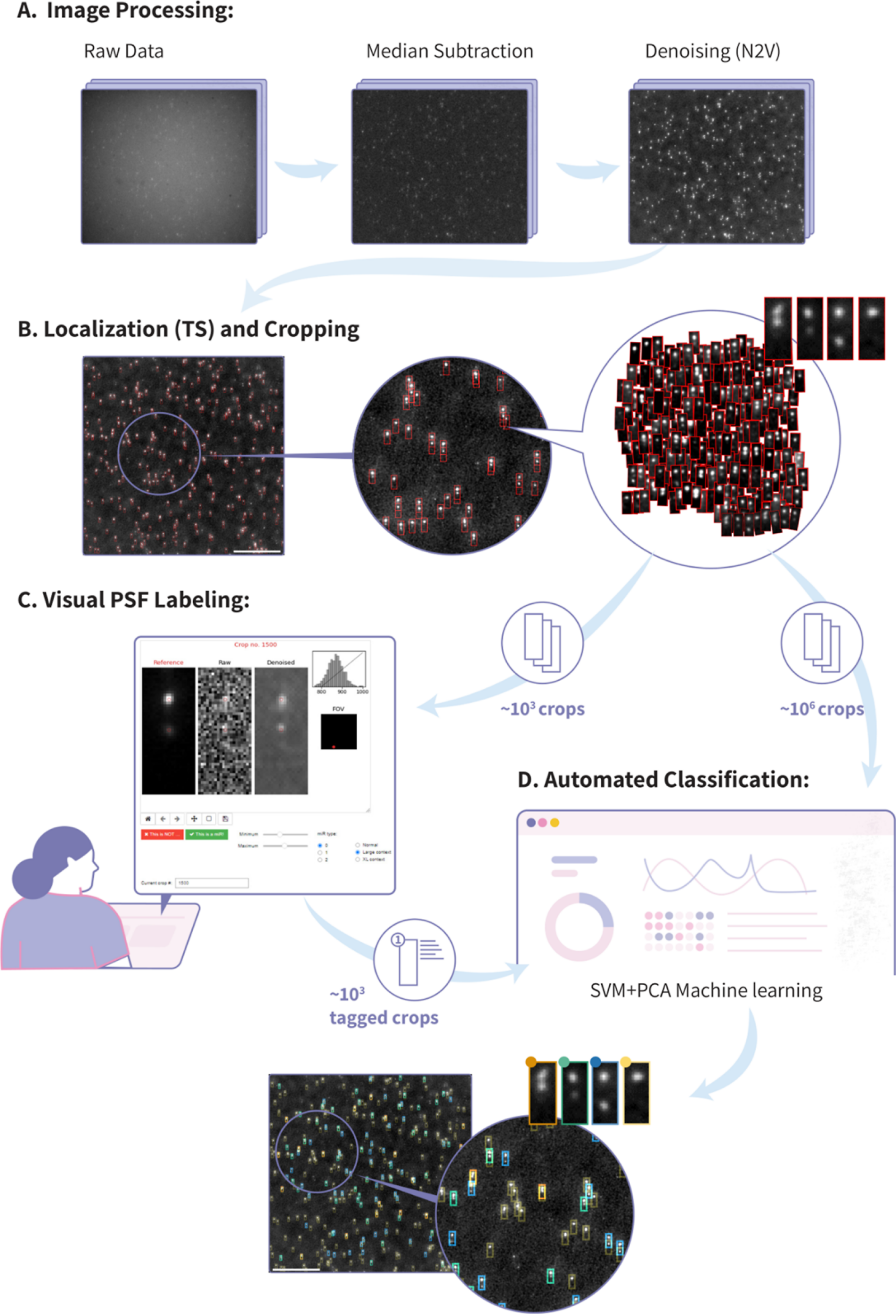
Automated multiplexed miR detection and classification at single-molecule resolution. (A) Before detecting and classifying the probe’s PSFs, three steps of image preprocessing are performed. First, using a multi-FOV stack, we calculate and subtract the pixel-wise median value from the raw images to remove the constant excitation background. Next, we further process the images to enhance the signal by using the deep learning-based Noise2Void (N2V) algorithm. (B) Denoised images are input to the ThunderSTORM (TS) localization plugin to detect all Gaussian spots and to crop 24 × 10 pixels rectangles around each Gaussian spot for further analysis (example crops are shown on the right). (C) Single miR-type samples are used for generating a training set for the automatic classifier. A small subset of ∼1000 crops are visually inspected by a user in V-TIMDER, a dedicated graphical user interface. The user visually tags each crop as a valid miR PSF or as noise. (D) Tagged crops gathered from three single miR-type samples are computationally mixed and used for training a machine-learning classifier model using SVM combined with PCA. The classifier is used to automatically classify mixed miR samples according to their PSFs: three different miR types (orange, green, and blue) or noise (yellow). Scale bars on FOV images in parts B and D are 30 μm.

First, the raw image stacks are preprocessed to enhance the SNR for subsequent single-molecule detection and classification (Figure 2A). The preprocessing protocol includes a standard background subtraction method utilizing a pixel-median calculation across the entire multi-FOV stack to eliminate spatial background dependencies. Subsequently, a deep learning-based technique known as Noise2Void (N2V)^38^ denoising is applied (see Methods and Table S3 for details), which effectively eliminates local zero-mean noise and further improves the quality of the single-molecule PSFs. This preprocessing workflow ensures optimal data quality and enhances the accuracy of the subsequent analysis and interpretation.

Next, to extract the relevant PSF data from the full FOV images, a cropping process is employed. This involves the localization of all two-dimensional diffraction-limited Gaussian shapes within the images using the ImageJ’s ThunderSTORM (TS) plugin^39^ (see Methods and Table S4 for details). Subsequently, a rectangular region measuring 24 × 10 pixels is cropped around each localization (Figure 2B). To ensure the optimal performance without compromising recall, the TS localization threshold is set as low as possible while excluding single-pixel localizations. It is noteworthy that since our spectral PSFs comprise two diffraction-limited Gaussian spots, the TS plugin detects each probe twice and thus rectangles are cropped around both the lower and upper spots. However, for downstream analysis, only the upper crop is pertinent as it encompasses the complete double-spot PSF (Figure 2B, rightmost crop examples). Consequently, it is anticipated that at least 50% of the generated crops will be classified as non-miR “noise”.

Finally, the crop classification and miR target expression profile reconstruction are performed by using an automated pipeline based on principal component analysis (PCA) and support vector machine (SVM) machine-learning. The PCA–SVM model takes crops as an input and assigns them to one of the three miR types, or categorizes them as noise when they do not exhibit a strong correspondence with any of the miR probes (see Methods and Table S5 for details). To train our model, small subsets containing ∼1500 crops from each of the three single miR species data sets were manually curated using Visual Tagging Interface for Machine-learning DEcision Refining (V-TIMDER), a custom Graphical User Interface (GUI) tool for PSF labeling. V-TIMDER enables users to visually label the crops as either “noise” or the relevant miR PSF, generating a labeled data set for the supervised machine-learning classifier. It provides various features that facilitate the users’ PSF classification, such as plotting the expected PSF image and overlaying the expected spots’ locations on the crop for easy visual comparison (Figure 2C top left, detailed interface description in Figure S9). The labeled results from V-TIMDER serve as training data for the automated PCA–SVM classifier, capable of classifying millions of crops from diverse miR distributions and mixtures (Figure 2C bottom).

To validate the results of the automated classifier on mixed samples, V-TIMDER was further adjusted to allow visual classification of such samples (Figure S9). In this “mixture” mode, rather than the binary choice between miR or noise for each crop as in the single-species case, the user has the flexibility to assign each crop to one of the three PSF types or classify it as noise.

**Figure 3:**
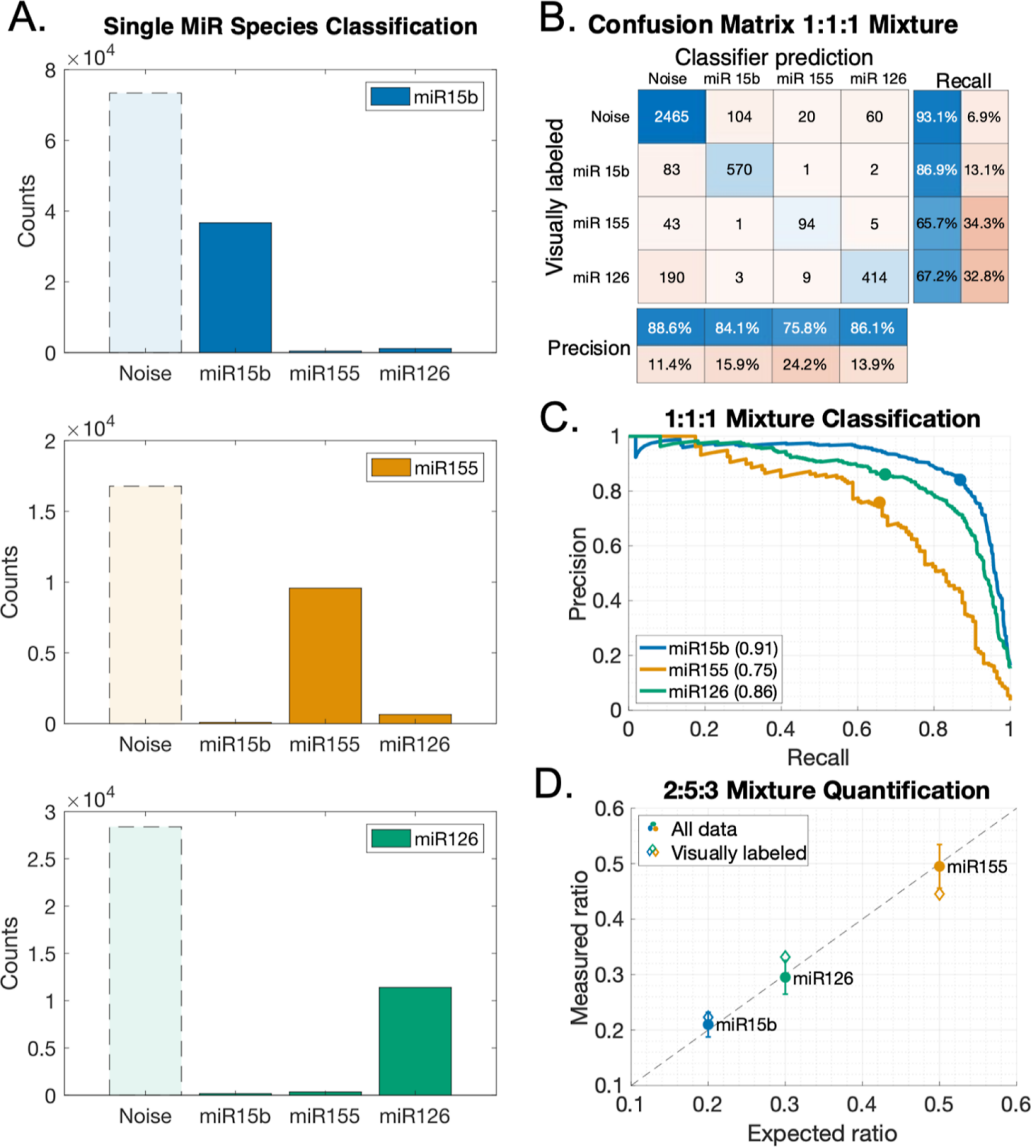
Experimental classification results. (A) Absolute count distributions of single miR-type samples automatically classified from 241 (miR 15b), 324 (miR 155), and 303 (miR 126) FOVs. The distributions show minimal cross-assignment of the classifier between miR types. Each panel shows the classified miR type for all crops taken from a sample containing only the type specified in the legend. (B) Confusion matrix for a mixed 1:1:1 miR sample (counted in 10 FOVs). The matrix compares the visually determined label to the automated classifier prediction. The matrix is predominantly diagonal, showing most classifier errors to be false noise classification and very little cross assignment between miR types. The precision and recall metrics are summarized for each class in the confusion matrix. (C) Precision and recall of each miR type as a function of the classification probability threshold. The area under the precision recall curve (AUC-PR) for the classification of each miR type is indicated in parentheses. Circles correspond to the precision and recall calculated from the confusion matrix in (B). (D) Determining the ratio of miR targets in an “unknown” synthetic mixed sample. The three synthetic miRs were mixed in a 2:5:3 ratio and were classified without supervision, and systematic errors were corrected by using the confusion matrix in panel (B). We were able to correctly resolve the underlying ratios of miRs (circles, total of 63,493 classified crops in 630 FOVs). The error bars represent the uncertainty of a 95% confidence interval. Diamonds represent the calculated ratio for a small subset of visually labeled crops (519 classified miR crops in five FOVs, ∼0.82% of the full data set).

The classified results are compiled to obtain the miR count distributions, which can be utilized for downstream analysis and future diagnostic applications. To benchmark the classifier performance, we analyzed the three different samples of a single miR-species individually, demonstrating the accurate classification capability of the automated classifier with minimal confusion among miR types (Figure 3A). Subsequently, we investigated two mixtures of the three miRs with ratios of 1:1:1 and 2:5:3 (miR-15b-5p, miR-155-5p, and miR-126-3p, respectively). To evaluate the classifier’s recall and precision capabilities, we utilized V-TIMDER to visually assess a small subset of crops taken from the 1:1:1 mixture’s data set (4064 visually labeled crops out of 197,963 classified crops). The labeled data were used as a test set to benchmark the performance of the automatic classifier on mixed samples. Comparison of visually labeled and classifier’s results is summarized with the confusion matrix in Figure 3B. The precision and recall for each miR type were evaluated from the matrix (marginals in Figure 3B and circles in Figure 3C). Evidently, the classifier predominately confounds between noise and the different classes and hardly mixes between miR types, allowing us to evaluate the performance of the classifier for each miR independently by performing a binary classification (see the Methods section) and obtaining precision-recall (PR) curves (Figure 3C). The PR curves and their corresponding area under the curve (AUC-PR)^40^ demonstrate a varying classification performance between miR targets, with the best performance for miR-15b-5p which has a more distinguished PSF. Essentially, the confusion matrix for the 1:1:1 mixture encompasses all the systematic classification errors. Thus, we use this equidistributed sample to correct our ratiometric readout for various experimental contributions such as different initial single-species concentrations (visible in Figure 3A), competitive binding effects which are known to affect the measured ratios,^31^ and minute PSF differences between the single-species and mixtures experiments due to the experimental focal changes (Figures 3A,B and S10 and S11). Since these physical effects skew the count distribution ratio in a constant manner, we can normalize the observed count distributions to estimate the true miR target ratios. By employing the inverted row-normalized confusion matrix on the classifier results and normalizing the result with the 1:1:1 absolute count distribution, we can effectively correct all inherent physical and classifier errors, obtaining a more precise representation of the miR mixtures’ underlying ratios (see the Methods section for details). We demonstrate this on a different mixture with a 2:5:3 ratio retrieving a very close result of a 2.10:4.95:2.95 ratio with our pipeline (Figure 3D). The confusion matrix also allows for assessing the overall errors of our pipeline (see the Methods section for details). Since the confusion matrix values are discrete measurements, we assumed that they have a multinomial noise distribution around the observed values (depicted in Figure 3B), allowing us to generate multiple confusion matrices and determine the miR ratio’s confidence bounds (Figure 3D, error bars correspond to two standard deviations or 95% of possible results. Further details are given in the Methods section). The evaluated ratio errors correspond well to the ratio results of a V-TIMDER visual classification benchmark test on a small subset of crops taken from the full data set (1565 crops out of 214,231 crops in the 2:5:3 data set, out of which 519 were visually assigned to one of the miR classes, and the rest were classified as noise). From this analysis, the dominant uncertainty in estimating the ratio distribution arises from misclassifications of miRs 126 and 155, while miR 15b is better classified in our model (see Figure S12 for the full distributions of simulated ratios). Nevertheless, according to this uncertainty estimation, in the worst-case scenario, we expect to distinguish between variations larger than 10% in mixture abundance ratios (Figure S12). It should be noted, however, that other error sources might be present and are not modeled by this analysis.

## Conclusions

Overall, our results demonstrate the capability of confidently detecting and reconstructing mixture distributions of three miR types simultaneously. Our simulations show that the method could be easily expanded to multiplex up to 11 types of miRs (Figure S8). MiRACLE’s multiplexing capabilities could be further enhanced by introducing 3- and 4-fluorophores probes, allowing us to combinatorically increase the uniquely resolvable spectral PSFs that can be classified by miRACLE [see Figure S13 showcasing 10 unique PSFs with only four fluorophore combinations, demonstrated using 100 nm colored silica beads (SBs)]. MiRacle provides single-molecule detection capability of unamplified miR targets with ultimate sensitivity. The method is fast, sensitive, and extremely cost-effective (<$4 per sample, Table S1), paving the way to robust profiling of small clinical panels of native miR and other RNA targets (see Figure S14 for a demonstration of multiplexed detection of two miR targets in RNA extracted from human plasma). Finally, the V-TIMDER tool developed here for visual spectral PSF classification could be readily implemented in a wider context of single-molecule applications involving PSF engineering.^41^

## Methods

### Slide Preparation

The miRs were captured on borosilicate glass coverslips (D 263 Schott glass, 75.5 × 25.5 mm^2^, ibidi GmbH, Germany), passivated with poly ethylene glycol (PEG). In brief, after cleaning, each coverslip was hydroxyl terminated with freshly prepared KOH. The coverslip was then pegylated with a mixture of Methoxy PEG Silane (mPEG-Silane MW5000, Laysan Bio Inc. AL, USA) and Biotin-PEG-Silane- (Biotin-PEG-Silane MW5000, Laysan Bio Inc. AL, USA) in a 1:100 stoichiometry in dehydrated HPLC grade ethanol. A six-channel ibidi sticky-slide (μ-Slide VI 0.4, ibidi GmbH, Germany) was mounted on top of the pegylated coverslip. Each channel was hydrated with 100 μL of DNase I and RNase-free deionized water for 15 min and then equilibrated with 100 μL of PBS for 30 min. Following this, each channel was activated with 100 μL of 1 nM monoclonal Anti-DNA-RNA hybrid S9.6 antibody diluted in PBS (S9.6, Mouse IgG2a kappa Isotype, conjugated with streptavidin, Ab01137–2.0, Absolute Antibody, Oxford, UK) to capture our targeted biomarkers. Following a 1 hour incubation with the antibody at room temperature, the channels were washed repeatedly with PBS to remove all excess unbound antibody. Thus, prepared channels were able to capture targeted miRs hybridized with labeled DNA probes as the DNA/RNA duplex (see the Supporting Information for details of the cleaning and passivation process).

### Synthetic miR Experiments

In all experiments with synthetic miRs and their complementary single-stranded DNA capture probes, 100 μL of the duplex at 50 pM concentration was used in each channel, which was preincubated with S9.6 antibody. After hybridization, a total of 31 μL of miR mixture was applied on the immobilized S9.6 antibody in one channel, incubated for 45 min, and washed 3× with PBS before imaging.

### Human Plasma Experiments

All small RNAs (including miRs) were purified from 500 μL of plasma using the miRNeasy Serum/Plasma Advanced Kit (QIAGEN GmbH, Hilden, Germany) in 15 μL of Rnase free water and stored at −20 °C. 15 μL of PBS was added to the purified RNA extract, following 0.1 fmol (1 μL of 5 pM) of miR-155-5p and miR-15b-5p capture probes (Figure 1C). The solution was left for 3 h hybridization at room temperature. After hybridization, a total of 31 μL of the solution was applied onto the preimmobilized S9.6 antibody slide, incubated for 45 min, and washed three times with PBS before imaging (results are shown in Figure S14).

### Multicolor SB Experiment

For each combination of fluorescent colors presented in Figure S13, a 5 μL of 100 nm SB functionalized with azide (Si100-AZ-1, Nanocs, NY, USA) was added to 100 μL of 1:1 ddH2O/ethanol solution and vortexed thoroughly. We then added to the solutions 0.2 μL of 10 mM AF-405, AF-488, AF-568, AF-647 DBCO conjugated dyes (AF647-DBCO, AF488-DBCO, AF405-DBCO, Jena Bioscience, Germany; AFDye 568-DBCO, Click Chemistry Tools, USA) according to the required combination of colors, followed by vigorous pipetting and vortex for creating homogenized distribution. The solution was left to incubate for 3 h at 37 °C after which the beads were cleaned from residual-free fluorophores by the following steps:

1. Centrifuging at 17,000 rpm at 4 °C for 15–30 min (or until a pellet is visible at the bottom of the tube. The pellet should be colored according to the dye used).
2. Gently removing the liquid while avoiding disturbing the pellet.
3. Adding 100 μL of 1:1 ddH_2_O/ethanol, pipetting vigorously, and vortexing for homogeneous distribution.
4. Repeating steps 1–3 four times. At the last repeat avoid step 3 and instead proceeding to step 5.
5. Suspending the washed pellet in 30 μL of ddH_2_O to obtain stock colored SB.

The stock solution was diluted 1:100 in ddH_2_O before imaging.

### Optical Setup

The optical setup was previously described elsewhere^35^ and is given here for the sake of completeness.

#### Excitation

For excitation, we used three lasers (Cobolt AB, Sweden) with wavelengths 488 nm (MLD 488, 200 mW max power), 561 nm (Jive 561, 500 mW max power), and 638 nm (MLD 638, 140 mW max power). All lasers were mounted on an in-house designed heatsink that coarse aligned their beam heights. Each laser beam was passed through a cleanup filter (LL01–488–12.5, LL01–561–12.5, LL01–638–12.5, Semrock, USA) and expanded to 12.5–20× its original diameter (3 × LB1157-A, 3 × LB1437-A, Thorlabs, USA). A motorized shutter (SH05, Thorlabs, USA) was used for modulating on/off the solid-state 561 nm laser, while the diode lasers were modulated directly on the laser head. The beams were then combined into a single beam using long-pass filters (Di03-R488-t1–25.4D, Di03-R561-t1–25.4D, Semrock, USA). To homogenize the excitation profile of the sample, the combined beam was passed through an identical setup to the one described in the work of Douglass et al.^42^ In short, the combined beam was injected into a compressing telescope (AC254–150-A-ML, AC254–050-A-ML, Thorlabs, USA) with a rotating diffuser (24–00066, Süss MicroOptics SA, Switzerland) placed ∼5 mm before the shared focal points of the telescope lenses. A series of six silver mirrors (PF10–03-P01, Thorlabs, USA) was then used to align the beam into a modified microscope frame (IX81, Olympus, Japan), through two identical microlense arrays (2 × MLA, 18–00201, Süss MicroOptics SA, Switzerland) separated by a distance equal to the microlense focal length and placed inside the microscope frame. The homogenized beam was reflected onto the objective lens (UPlanXApo 60X NA1.42, Olympus, Japan) by a four-band multichroic mirror (Di03-R405/488/532/635, Semrock, USA). The sample was placed on top of a motorized XYZ stage (MS-2000, ASI, USA) with an 890 nm light-emitting diode-based autofocus system (CRISP, ASI, USA), which enabled scanning through multiple fields of view.

#### Emission

The emitted fluorescence light was gathered by the same objective and transmitted through the multichroic mirror onto a standard Olympus tube lens to create an intermediate image at the exit of the microscope frame. This image was passed through a multiband emission filter (FF01–440/521/607/694/809–25, Semrock, USA) and was then directed into a magnifying telescope (Apo-Rodagon-N 105 mm, Qioptiq GmbH, Germany and Olympus’ wide field tube lens with 180 mm focal length, #36–401, Edmund Optics, USA), with two commercial direct vision prisms (117,240, Equascience, France) placed within the infinity space between the lenses and mounted on two motorized rotators (8MR190–2–28, Altechna UAB, Lithuania) controlling the prisms’ angles around the optical axis. The final image was acquired on a back illuminated sCMOS camera (Prime BSI, Teledyne Photometrics, USA).

Image acquisition was coordinated using micromanager software,^43^ controlling camera acquisition, laser excitation, XY stage location, and prism rotator angles. The camera and laser excitation were synchronized using an in-house-built TTL controller based on an Arduino Uno board (Arduino AG, Italy).

### Image Acquisition

The sample lanes were scanned laterally and imaged with a single acquisition per FOV, obtaining ∼2000 FOVs per sample. The different fluorophores used in our probe designs have different photophysical properties, effecting their overall brightness. Therefore, since all lasers excite the probes simultaneously, we adjusted individual laser intensities to achieve homogeneous intensity profiles of the probes’ PSFs.

For imaging, we used a single exposure per FOV with a relatively long exposure time (800 ms per frame) compared to standard multiframe fluorescence imaging (30–100 ms per frame). However, this long exposure did not contribute to extensive photobleaching as excitation power was distributed over a large field of illumination (130 × 130 μm^2^), resulting in relatively low irradiation at the sample (∼0.2 kW/cm^2^). Furthermore, even if a fluorophore did bleach during the single-frame acquisition, its signal was still recorded during this exposure, resulting in optimized probe detection and SNR.

The same optimization was performed in the colored SB experiment using lower laser powers to excite the higher fluorophore densities found on the beads. The acquisition parameters are described in Table 1.

**Table 1:** Image Acquisition Parameters^a^

experimental target	excitation	RPA	emission filter	exposure time (ms)	laser intensities at laser output
synthetic miRs	all lasers simultaneously	177.5	FF01–440/521/607/694/809–25	800	638 laser–90 mW
					561 laser–90 mW
					488 laser–160 mW
					
colored 100 nm SB	all lasers simultaneously	178	NF03–405/488/561/635E-25	250	638 laser–30 mW
					561 laser–10 mW
					488 laser–40 mW
					405 laser–60 mW
					
	sequential laser excitation	178	NF03–405/488/561/635E-25	250	638 laser–30 mW
					561 laser–10 mW
					488 laser–40 mW
					405 laser–60 mW

a RPA—relative prism angle.

### Image Processing

The image processing scheme used for generating miR distributions from the raw dispersed images was divided into four subprocesses: (i) image preprocessing, (ii) PSF detection, (iii) PSF visual labeling with V-TIMDER for training the automatic classifier, and (iv) automatic PSF classification.

#### Image Pre-Processing

Before the miRs were analyzed, the images were processed in the following way in order to improve the SNR:

1. First, the inhomogeneous background in each FOV was removed by subtracting a pixel-wise median calculated across all FOVs in the experiment.
2. Outlying FOVs were pruned based on a rough statistical measure: keeping only the FOVs whose mean of the (median-subtracted) positive pixels and the mean of the negative pixels are smaller in absolute value than some threshold. This filters out FOVs with extreme features such as bubbles, and FOVs with a background that did not agree with the median. The threshold is chosen so about 80% of the FOVs are kept.
3. We then used Noise2Void (N2V), a self-supervised deep learning algorithm to denoise the images and improve the SNR. The main assumption of the algorithm is that the noise is pixel-wise independent, an assumption that holds for the pruned, median-subtracted images. The hyperparameters for the N2V model are detailed in the Supporting Information (Table S3).

#### PSF Detection

1. We used FIJI’s ThunderSTORM plugin^39^ to find blobs (peaks) in the denoised images. This provides the *x*,*y* coordinates of each blob in each FOV. The thresholds were selected such that all the top blobs of all miRs in the image were detected, in addition to “noise blobs” which are blobs that are not part of a miR, or the bottom blob of a miR. Threshold selection was carried out based on thorough visual inspection of a few randomly selected FOVs.
2. We then merged blobs that are very close to each other in the same FOV into a single blob at their mean position. The distance threshold below which blobs are merged is significantly smaller than the distance between miRs. Blobs very close to the FOV boundary were discarded.
3. We took rectangular crops around each blob in both the noisy (median-subtracted) and denoised images. The dimensions of the crops, 24 × 10 pixels, were selected such that both top and bottom blobs appear in each miR crop. For more details, see note 6 in the Supporting Information.

#### Visual PSF Labeling Using V-TIMDER

1. To empirically estimate each miR’s PSF from the data (Figure S15), we calculated the pixel-wise median of the single miR species over ∼10^5^ noisy crops. These empirical PSFs have an excellent SNR with two distinct blobs.
2. To generate training, validation, and test data sets for our machine-learning model, we randomly choose a few thousands of blobs taken from a small (∼5–10) number of FOVs. For these blobs, in addition to the standard crops, larger crops (×2 and ×10 the standard crop size, see Figures S9 and S15) from both the noisy and denoised images are taken to provide a better visual context of the blobs. We feed this ensemble of crops, as well as the empirical PSFs to V-TIMDER, a custom-built GUI that allows convenient visual classification of the crops. This GUI has two modes: a “binary” mode where the user needs to determine for each blob whether it is the top blob of a miR or not, and a “mixture” mode where the user also determines which miR species it belongs to. The binary mode is used for the single-species data sets, and the mixture mode is used for the mixture data sets. In this work, we visually classified 1701 (miR 15b), 1783 (miR 155), and 1795 (miR 126) crops from the single-species data sets, and another 4064 and 1565 crops were classified from the 1:1:1 and 2:5:3 mixtures, respectively.

#### Automatic PSF Classification Using Machine Learning

The full classifier pipeline is provided in Figure S16

1. Classifier training and validation: for the training process, we used 90% of the denoised crops from the single-species visually labeled data set. The remaining 10% were used for model validation, obtaining metrics of the classifier performance and hyperparameter tuning. The training was carried out for both classifier’s modules with preceding data augmentation and preprocessing steps:
  a. Data augmentation: the training data set was augmented by adding multiple realizations of a weak random pixel-wise Gaussian noise to each denoised crop labeled as miR (see Figure S17 for example augmentations and Table S5 for details). We found this step to be crucial for the classifier’s success. This also increased our training data set size by a factor of 3.
  b. Crops preprocessing: all crops were preprocessed by the following pipeline:
    i. Background subtraction: for each denoised crop, subtract the median of the pixels at the crop edges. If this causes any of the four central pixels of the top blob to become negative, discard the crop. Otherwise, replace negative pixels with zero values.
    ii. Normalization: normalize each crop by a factor k such that the four central pixels of the top blob have a mean of 1 (see Table S5 for details). The normalization constant *k* is saved for future use (item d below).
    iii. Symmetrization: add the horizontally flipped mirror image of the crop to itself. This produces a symmetrical crop. We keep only the right half, reducing the crop size from 240 pixels to 120.
  c. Unsupervised PCA training: the preprocessed symmetrized crops from the single-species visually labeled data set were fed into an unsupervised PCA training procedure, extracting the 20 most significant components characterizing the data set to be later used for dimensionality reduction (see Figure S18 for learnt PCA components).
  d. Preprocessing before SVM classification: the symmetrized crops together with their saved *k* factor were further processed before being fed to the SVM classifier:
    i. Dimensionality reduction: project the symmetrized crop on the learnt 20 PCA components to obtain 20 coefficients.
    ii. Standardization: normalize the 20 coefficients as well as *k* to have a zero mean and a unit variance.
  e. SVM training: the visually labeled single-species data sets’ standardized PCA coefficients, *k* values, and labels were fed to a RBF-kernel SVM classifier^44,45^ (see Table S5 for classifier’s details). The classifier chooses between four classes, representing the three miR species and a noise class for crops that do not contain a miR (either the bottom spot in a miR or a false detection from ThunderSTORM). The resulting SVM model was saved for further use.
  f. SVM classification: for the validation and test data, the learnt SVM model was directly applied on the standardized PCA coefficients and *k* values to provide classification. The final classification by the classifier can be carried out in two ways:
    i. Classification using Scikit-Learn’s *predict* method,^46^ which returns the predicted class. This method was used for all practical purposes including training, validation, testing, and classifying (as displayed in Figures 3A,B,D, S19 and S20).
    ii. Probability prediction using Scikit-Learn’s *predict_proba* method,^47^ which returns a probability vector of length four, describing the model’s estimation of the probability that the crop belongs to each of the classes. This method was used to generate the PR curves (Figure 3C).
  g. Validation: the performance of the trained model was validated on 10% of the labeled data set (see the confusion matrix in Figure S19). The validation crops processing was performed according to steps b and d.
2. Deployment: The 1:1:1 and the 2:5:3 mixture data sets, as well as the unlabeled subsets of the three single-species data sets, were fed through the above pipeline (steps 1b, 1d, and 1f) for the purposes of calibration and testing, as detailed below.

### Precision and Recall

We use the labeled subset of the 1:1:1 mixture both for evaluating the model’s performance (with respect to the V-TIMDER true labels) and for calibrating the classifier. Using the classifier on this subset, we obtain a 4 × 4 confusion matrix *C*_*ij*_ (Figure 3B) whose entries are the number of blobs whose true label (according to V-TIMDER) is *i* and were predicted to be in class *j*. The matrix *C* is used for both performance evaluation and model calibration.

The recall for class *i*, *r*_*i*_ is the fraction of the true occurrences of this class that were correctly detected, *r*_*i*_ = *C*_*ii*_/∑_*j*_C_*ij*_. Similarly, the precision *p_j_* the fraction of blobs classified as class *j*, whose true class is indeed class *j*, is *p*_*j*_ = *C*_*jj*_/∑_*i*_C_*ij*_. The circle markers in Figure 3C are the precision and recall values for each miR class.

A more descriptive metric of the classification strength is the PR curves for the 1:1:1 subset, as shown in Figure 3C. These curves are generated by using the classifier’s probability prediction method, in which the model outputs a probability vector *P*_*i*_, corresponding to the predicted probability that the sample belongs to class *i*. For each of the three miR classes, we performed a binary classification for that miR class by thresholding *P*_*i*_. The threshold is varied between 0 and 1, and for each threshold, we calculate the binary classification’s precision and recall.

### Calibration and Testing

For each data set, the classifier returns a vector of length 4, which we denote by *h*, corresponding to the predicted counts of occurrences in each class. The relation between *h*, and the true counts of these classes, *h*′, is given by definition, by *h* = *Ĉh*′, where *Ĉ* is the row-normalized confusion matrix *Ĉ*_*ij*_ = *C*_*ij*_/∑_*k*_*C*_*kj*_. Therefore, to obtain the best estimate of true counts, we multiply the predicted class counts *h* by the inverse of *Ĉ*. This gives the final estimation of the abundance of each class.

Doing this procedure for each of the unlabeled 1:1:1 and 2:5:3 mixture data sets, we obtain the estimation for the counts, *h*_111_′ and *h*_253_′. The element-wise division *h*_253_′/*h*_111_′ gives our estimate for the miR ratios in the 2:5:3 mixed data set. This division accounts for experimental effects, as mentioned in the text, such as competitive binding of different miR types and PSF variations. This estimate, excluding its noise class, was normalized so its three remaining entries sum to 1, as illustrated in Figure 3D.

## Uncertainty Estimation

To estimate our prediction uncertainty, we repeated the above procedure but with noise added to confusion matrix *C* and counts vectors *h*_111_ and *h*_253_. Specifically, we replaced *C* with a pair of matrices drawn from a multinomial distribution whose mean value is *C*, and replaced the counts *h*_111_ and *h*_253_ with counts drawn from Gaussian distributions with means equal to *h*_111_ and *h*_253_ and standard deviations of 5% of the means. Each matrix from the pair of randomized matrices was inverted to “unconfuse” the randomized count vectors, and the resulting *h*_111_′ and *h*_253_′ were divided and normalized to obtain a miR ratio vector. We repeated this randomization procedure 10,000 times to obtain an ensemble of ratio vectors (see Figure S12). We then fit a 2D Gaussian to this ensemble to obtain the mean and the covariance matrix. The ratios in Figure 3D correspond to the mean vector projected onto the single-species vectors on the simplex, and the errors correspond to a 95% confidence interval (two standard deviations) for each miR type, calculated by projecting the covariance matrix onto the single-species vectors.

## PSF Simulations

All simulations were performed by a home-built Matlab code. Here, we provide a short description of the pipeline:

1. Excitation and emission spectra of 16 commercial fluorophores together with our four-notch filter were downloaded from Semrock’s SearchLight spectra viewer (names of fluorophores are provided in Table S2). Each of the fluorophore’s spectrum was multiplied by the filter’s spectrum to produce the actual spectrum visible on our camera.
2. The wavelength to pixel displacement calibration curve of our CoCoS setup (which was calculated previously^35^ was adjusted according to the experimentally used relative prism angle (RPA) by multiplying the entire curve by sin((180-RPA)/2).
3. Chosen double–fluorophore combinations were then simulated by converting each fluorophore’s spectrum into a diffraction-limited dispersed image. This was performed by assigning a Gaussian with unity amplitude and 1.15 pixel standard deviation to each wavelength in the emission spectrum. Each Gaussian was displaced according to the RPA-adjusted displacement curve and summed together with other Gaussians. Finally, the total summed intensity of all Gaussians was normalized to unity and multiplied by an excitation efficiency factor which was calculated by the excitation spectrum value (fractions only) at the excitation laser wavelength.
4. This process was repeated for the second fluorophore, and both images were summed to provide the dual-fluorophore spectral image.
5. When needed, a noise model was added to the simulated dual-fluorophore spectral PSF image using the “imnoise” function in Matlab. The noise model used in this work was a sum of a Poisson distributed shot-noise and Gaussian noise with a constant mean of 0.3 and a changing variance (see Figure S8).

## Data Availability

The raw data, python, and Matlab codes have been deposited to the Ebenstein group’s GitHub page (https://github.com/ebensteinLab/miRacle_V0).

## Supporting Information Available

Additional experimental details and methods, including images of control experiments, simulations, and example V-TIMDER GUI (PDF)
