# Supplementary material for: Machine-Learning-Based Single-Molecule Quantification of Circulating MicroRNA Mixtures
Source: ACS Sens. 2023 Oct 4;8(10):3781–92. doi: 10.1021/acssensors.3c01234 (PMC10616852; doi:10.1021/acssensors.3c01234)
Supplement: Supplementary file 1 — se3c01234_si_001.pdf [file se3c01234_si_001.pdf]

Supporting Information: Machine-Learning-Based Single-Molecule Quantification of Circulating MicroRNA Mixtures

Jonathan Jeffet[1,2,4†], Sayan Mondal[2,4†], Amit Federbush[1,5†], Nadav Tenenboim[1,2,4], Miriam Neaman[2,6], Jasline Deek[2], Yuval Ebenstein[2,3,4,7] *, Yohai Bar-Sinai[1,5,7]*

1. School of Physics and Astronomy, Raymond and Beverly Sackler Faculty of Exact Sciences, Tel Aviv University, Tel Aviv 6997801, Israel
2. School of Chemistry, Raymond and Beverly Sackler Faculty of Exact Sciences, Tel Aviv University, Tel Aviv 6997801, Israel
3. Department of Biomedical Engineering, Fleischman Faculty of Engineering, Tel Aviv University, Tel Aviv, 6997801, Israel
4. Center for Nanoscience and Nanotechnology, Tel Aviv University, Tel Aviv 6997801, Israel
5. The Center for Physics and Chemistry of Living Systems, Tel Aviv University, Tel Aviv, 6997801, Israel
6. Department of Hematology, Tel Aviv Sourasky Medical Center, Tel Aviv, Israel
7. Center for AI & Data Science (TAD), Tel Aviv University, Tel Aviv, 6997801, Israel

† Equal contribution
*Corresponding author: ybarsinai@gmail.com

1. <u>Cost of goods analysis (July 2023)</u>

Material	Amount	Cost ($)	Amount needed /Sample	Total No. of Samples	Cost/Sample ($)
Mirneasy plasma advanced kit - Qiagen	*50 preps*	*$750*	*1 prep*	*50*	*$15.00*
**5 Reporter probes**	0.25 nMol Guaranteed	$9,750	0.1 fMol/Sample	2500000	$0.0039
**S9.6 Antibody (1 mg/ml)**	100 ul, 6.8 uM	$498	60 pM *60 uL	167,000	$0.0030
**Coverslip (Ibidi)**	100	$171	6 samples/slide	600	$0.29
**Sticky Slide**	15 Slides, 6 Channel/Slide	$261	1 ch/ sample	90	$2.88
**mPEG NHS**	5 g	$504	50 mg/20 slides	12000	$0.042
**Biotin-PEG-SVA**	1 g	$504	0.5 mg/20 slides	240000	$0.0021
				Total price per sample :	$3.22

Table S 1 - Costs per sample for multiplexed detection of 5 miR reporters. Cost analysis is excluding the RNA extraction kit as this step can be skipped in future implementations.

2. Reporter probe sequences:

- Capture probe for hsa-miR-15b-5p (ATTO488-ATTO647N):

/5ATTO488K/TT AGT TGT AAA CCA TGA TGT GCT GCT AAT GTA /3ATTO647NN/

- Capture probe for hsa-miR-155-5p (AF546-ATTO647N):

/5Alex546N/TT AGT AAC CCC TAT CAC GAT TAG CAT TAA ATG TA/3ATTO647NK/

- Capture probe for hsa-miR-126-3p (ATTO488-ATTO565N):

/5ATTO488N/AC TTA GTC GCA TTA TTA CTC ACG GTA CGA ATG TAT C/3ATTO565N/

3. <u>Control experiments:</u>

a. <u>Surface PEG passivation characterization</u>

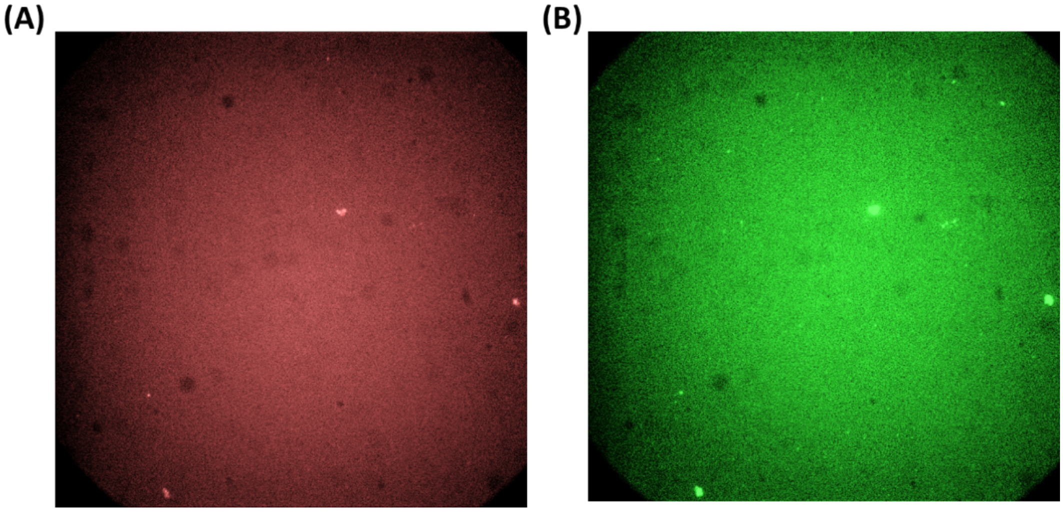

Figure S1 - Fluorescently labeled single-stranded DNA (ssDNA) capture probe does not non-specifically bind to Peg and Peg-biotin passivated surfaces. 100 ul of 100 pM ssDNA labeled with Alexa 647 (ssDNA-A647) was incubated on peg-biotinylated (Peg:Peg-Biotin = 100:1) glass surface for one hour, then washed 3 times with 100 ul PBS before imaging, and a field of view (FOV) with (A) 633 nm and with (B) 561 nm laser is shown. The FOV with 633 nm excitation shows that the ssDNA-A647 probe does not stick on the passivated surface and gets washed effectively with 3x gentle washing. The bright particles in (A) are not ssDNA-A647, and are other impurities seen in (B) with 561 nm laser excitation.

b. <u>S 9.6 non-specific ssDNA capture control</u>

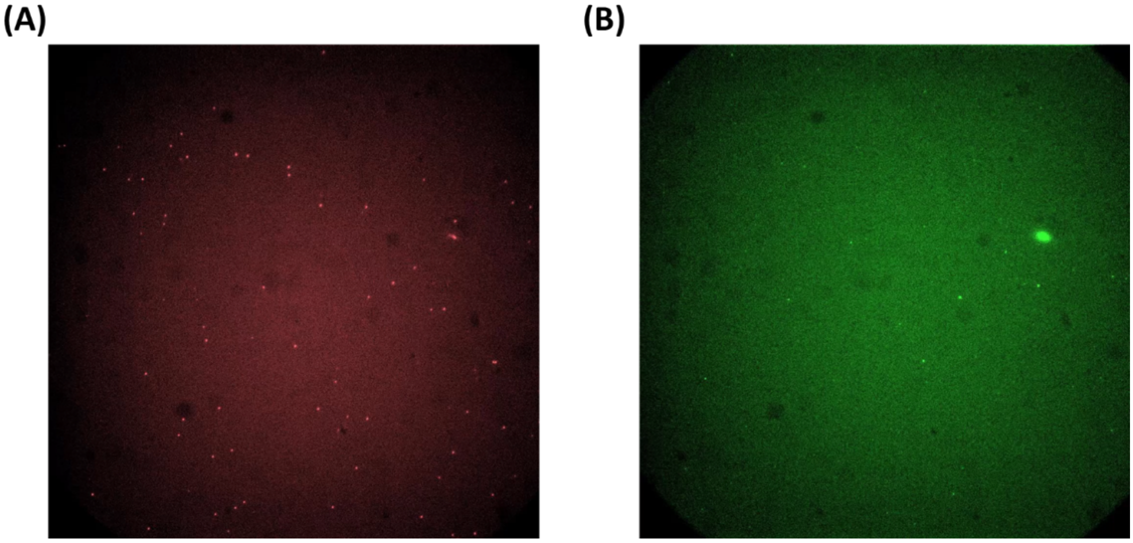

Figure S2 - ssDNA capture probes partially bind to S9.6 anti-DNA:RNA hybrid antibody. 100 ul of 100 pM ssDNA labeled with Alexa 647 (ssDNA-A647) was incubated on peg-biotinylated (Peg:Peg-Biotin = 100:1) glass surface with immobilized S9.6 antibody for one hour, then washed 3 times with 100 ul PBS before imaging, and a field of view (FOV) with (A) 633 nm and with (B) 561 nm laser is shown. The FOV with 633 nm excitation shows that the ssDNA-A647 probes indeed partially bind to S9.6-immobilized and PEG passivated surfaces. The bright particles in (A) are ssDNA capture probes, whereas very few bright spots seen in (B) with 561 nm laser excitation confirm that the single particles seen in (A) are indeed ssDNA-A647 capture probes.

c. S 9.6 non-specific miR capture control

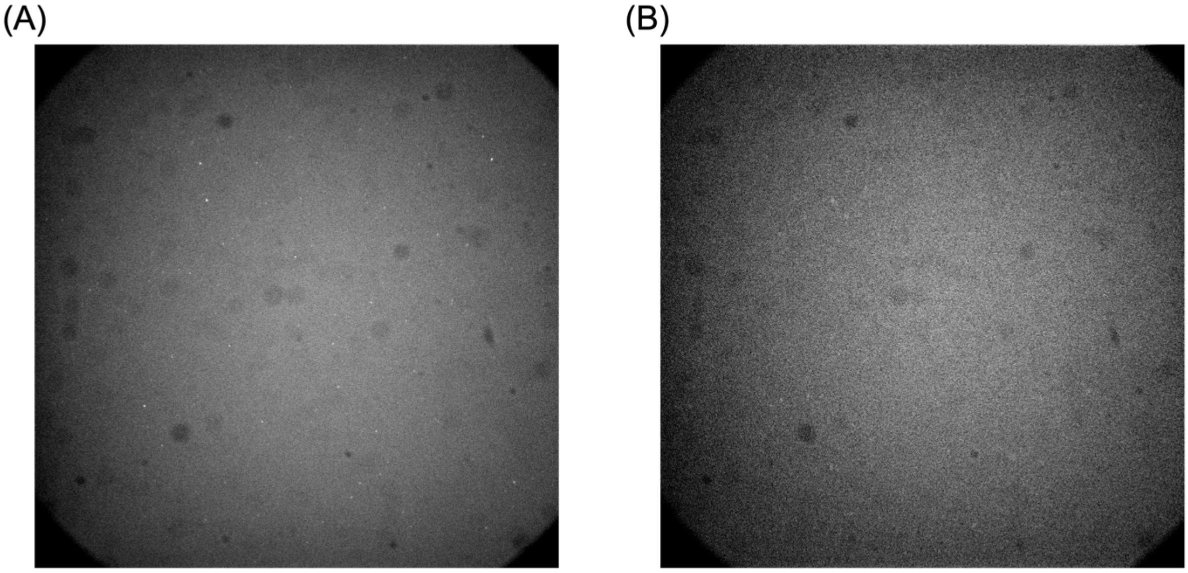

Figure S3 - S9.6 anti-DNA:RNA hybrid antibody does not capture single-stranded microRNAs. 100 ul of 100 pM synthetic miR (hsa-mir-15b-5p) labeled with Alexa 488 (miR-A488) was incubated on peg-biotinylated (Peg:Peg-Biotin = 100:1) glass surface with immobilized S9.6 antibody for one hour, then washed the 3 times with 100 ul PBS before imaging, and a field of view (FOV) with (A) 488 nm and with (B) 561 nm laser is shown. The FOV with 633 nm excitation shows that the synthetic miR-A488 has a small affinity for binding to immobilized S9.6 antibody and PEG passivated surface.

d. S 9.6 specific miR:Probe capture control

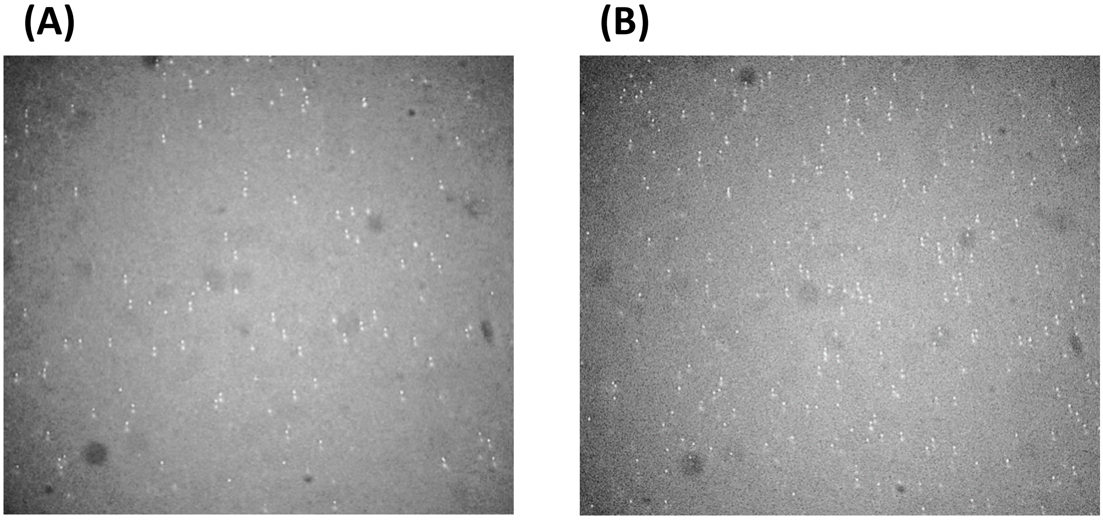

Figure S4 - S9.6 anti-DNA:RNA hybrid antibody specifically captures DNA:RNA duplex, and does not catch either ss-miR or ss-DNA capture probes in presence of DNA:RNA duplexes. CoCoS image with a single exposure of 800 ms with both 488 and 633 nm excitation, and at RPA 175 for optimal detection of dispersed emission. ssDNA-A647 capture probe is in (A) one time excess, and (B) seven times excess to DNA:RNA hybrids while incubating on immobilized S9.6 antibody, and then washed 3x with PBS before imaging. The DNA:RNA hybrids are detected as doubly labeled single molecules as ss-DNA capture probe is labeled with Alexa 647, and the target hsa-miR-15b-5p is labeled with AF488. (see Experimental methods for more details) In both (A) and (B) more than 98% detectable signal originates from duplex (two blobs separated by 12 pixels), and singly labeled ss-DNA or hsa-miR-15b-5p are not observed.

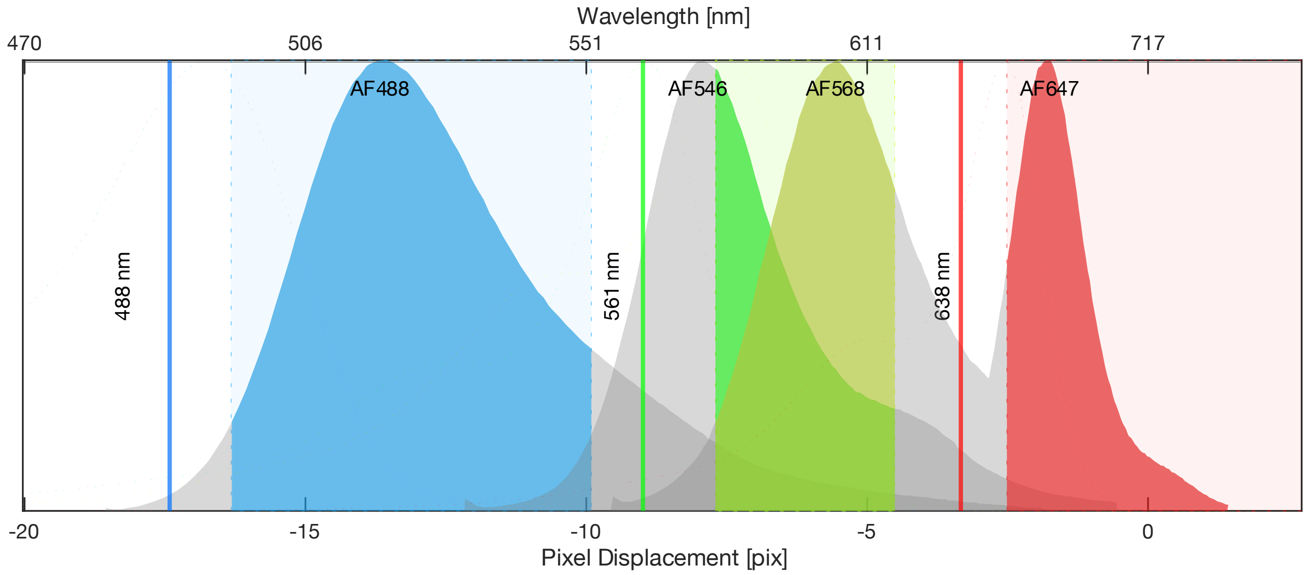

Figure S5 – Theoretical emission spectra of the four fluorophores used for the three probes design. Colored patches indicate the multi-band emission filter channels (Table 1 in the main text). The three lasers used for exciting the four fluorophores are displayed as solid vertical lines. The plot is stretched according to the non-linear dispersion curve of the optical system (Figure S6), showing the theoretical pixel displacement (bottom X-axis) of each wavelength (top X-axis) in the fluorophores' spectra.

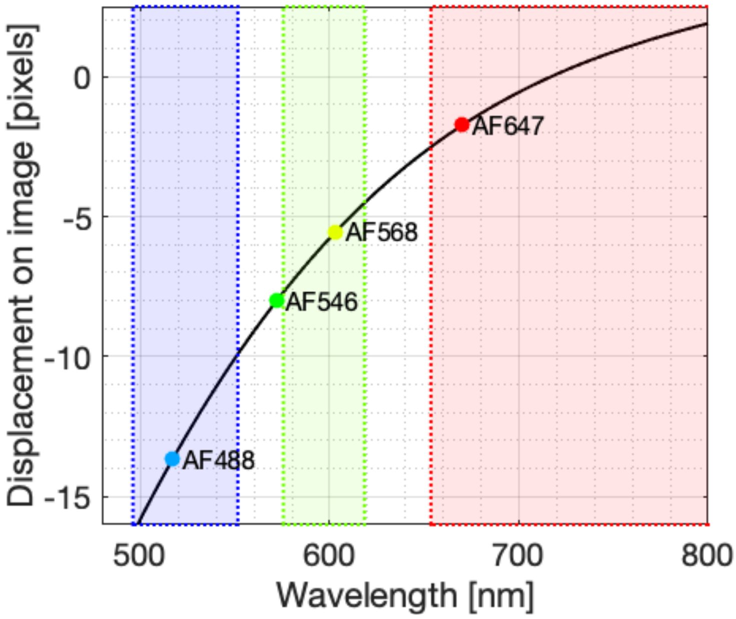

Figure S6 – Dispersion curve of the CoCoS setup using relative prism angle (RPA) of 177.5. The dispersion curve was experimentally extracted as previously described in ref[1]*. The colored circles correspond to the maximal emission wavelengths of the fluorophores used for designing the three probes used in the experiment, while the colored patches stand for the multi-band filter's transmission channels.*

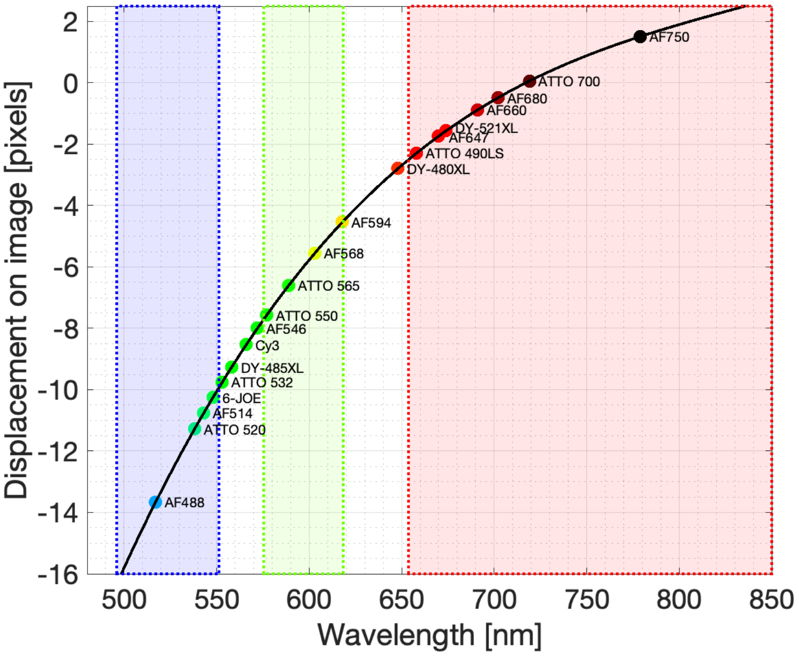

Figure S7 – Same curve as in Figure S6 with maximal emission wavelengths of all fluorophores used for simulating PSFs of optional combinations for future probes.

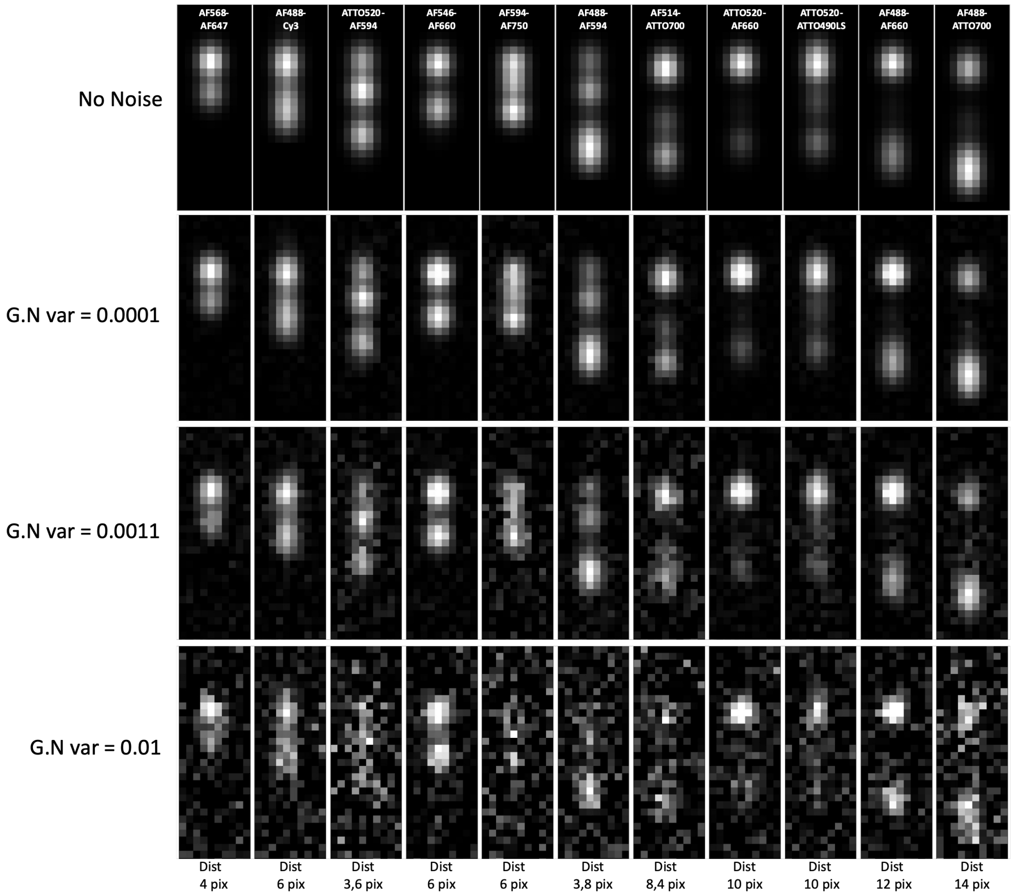

Figure S8 - PSF simulations with varying noise distributions. 11 fluorophore-pairs with distinct PSFs were simulated in Matlab according to the fluorophores' spectra and the dispersion profile of CoCoS with RPA=177.5. Each row corresponds to simulated Gaussian noise (G.N) added over the same PSF data as in the first row. The G.N is distributed around 0.3 mean and with variance as depicted to the left (corresponding roughly to SNR of ~25, 7 and 1.7, from top to bottom). Poisson distributed noise was added to all noise cases. As can be seen, the Gaussian noise which corresponds to the fluorescence background and pixel readout noises is the main limiting factor for efficient classification of multiple miR targets.

4. Names and parameters of fluorophores used for simulations

Name	AF 488	ATTO 520	AF 514	6-JOE	Cy3	AF 546	ATTO 550	AF 568	AF 594	ATTO 490LS	AF 647	AF 660	AF 680	ATTO 700	AF 700	AF 750
Ex. max (nm)	493	496	517	519	520	554	554	561	579	590	653	663	679	696	700	752
Em. Max (nm)	517	538	543	548	566	572	577	603	618	658	670	691	702	719	719	779
Ex. Factor	0.94	0.42	0.43	0.38	0.93	1.09	0.90	0.69	0.54	1.04	0.79	1.15	0.74	0.44	0.56	0.22

Table S 2 - Fluorophores used for PSFs simulations. The fluorophores' peak excitation and emission wavelengths are given together with their calculated excitation factor (sum of all excitation spectrum values at 488, 561, and 640 nm laser lines).

5. **Software parameters**

**N2V Parameter**	**Value**
unet_kern_size	3
train_loss	mae
batch_norm	True
train_batch_size	128
n2v_perc_pix	0.198
n2v_patch_shape	(64,64)
n2v_neighborhood_radius	3
unet_residual	False
n2v_manipulator	median
blurpool	True
skipone	False
train_steps_per_epoch	$N_{patches}/128$

Table S3 - N2V configuration parameters (N2V Version: 0.3.2). We used 250 randomly selected fields-of-view (FOVs), from an artificial mix of all the single species datasets (with equal proportions to each miR dataset). From these images, the N2V method of generate_patches_from_list generated patches to train the model. We trained our model for 100 epochs, although about 20 epochs also seemed to suffice.

ThunderSTORM category	Parameter	Value
Camera setup	Pixel size [nm]	120
	Photoelectrons per A/D count	3.6
	Base level [A/D counts]	0
	EM gain	Unchecked
Image filtering	Filter	Wavelet filter (B-Spline)
	B-Spline order	3
	B-Spline scale	1.7
Approximate localization of molecules	Method	Local maximum
	Peak intensity threshold	**20***
	Connectivity	8-neighborhood
Sub-pixel localization of molecules	Method	PSF: Integrated Gaussian
	Fitting radius [px]	3
	Fitting method	Weighted Least Squares
	Initial sigma [px]	1.6
	Multi-emitter fitting analysis	Disabled

Table S4 - ThunderSTORM configuration parameters (TS version: 1.3).
** This is an important parameter that could potentially change for different experiments according to the sample-specific SNR and noise distribution. The peak intensity threshold was selected by visual trial and error to ensure all miRs are detected, even at the cost of adding many false detections (due to the classifier's ability to remove them in the next processing steps, see methods)*

Augmentation	Number of augmented crops	×6 the number of visually-labeled denoised miR crops (omitting crops labeled as noise), added to the original crops
	Augmentation strength	Gaussian noise with mean zero and standard deviation of 15
Normalization	Normalization type	Normalize the entire crop so the entries of pixels (7,5),(7,6),(8,5),(8,6) have a mean of 1 (counting pixels from 1).
PCA	Number of components	20
SVM	Kernel	RBF
	Inverse regularization C	0.05

Table S5 - Classification pipeline parameters (sklearn version 1.1.3). SVM was trained using sklearn.svm.SVC and PCA was trained using sklearn.decomposition.PCA.

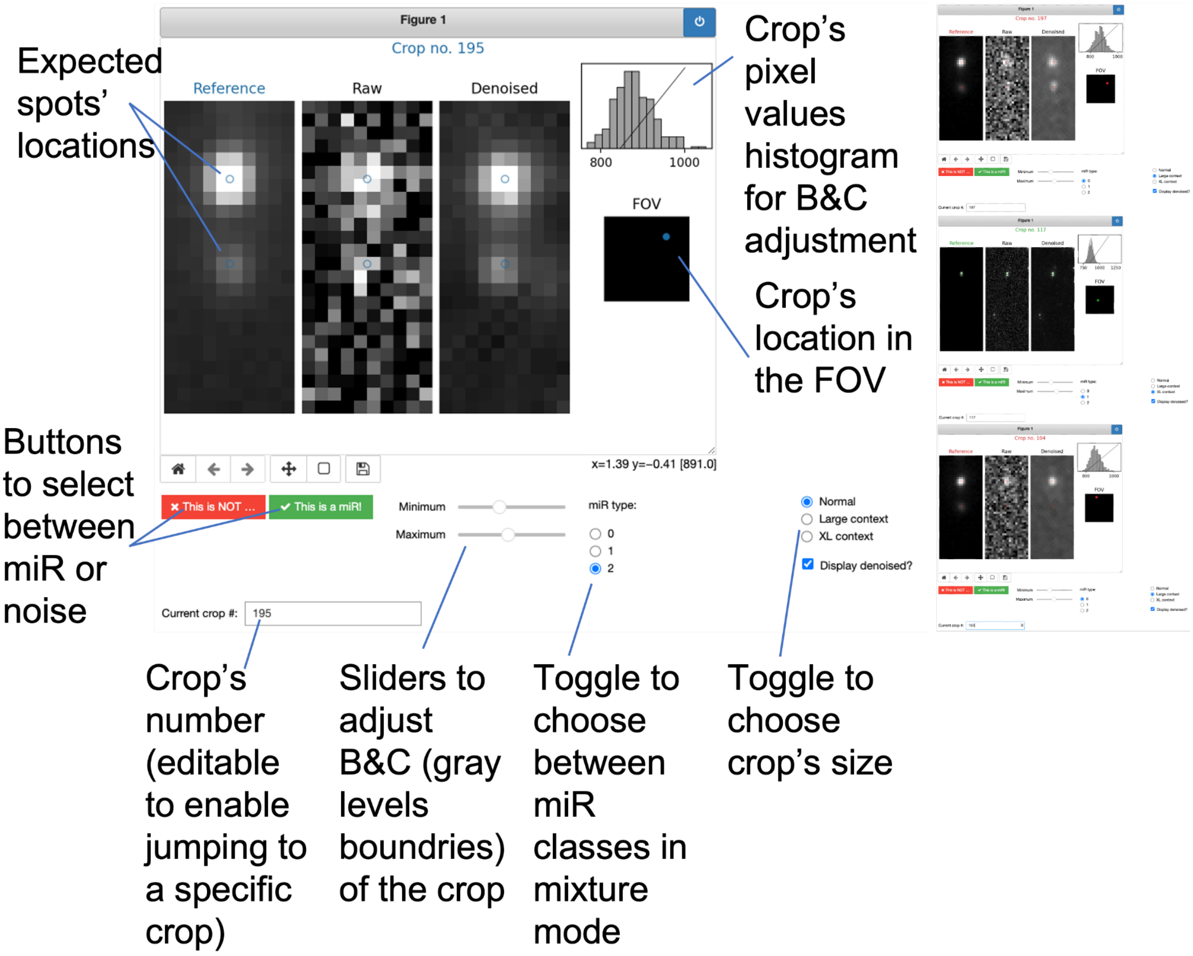

Figure S9 - V-TIMDER visual explanation. Left, the V-TIMDER GUI in "mixture" mode explained on a miR-126 PSF in normal context crop size (24X10 pixels). In "binary" mode the miR type toggle is removed. Right, examples for a large context (48X20 pixels) crop of miR-15b PSF (top), extra-large context (240X100 pixels) crop of miR-155 (center), and large context of a single spot, noise crop (bottom).

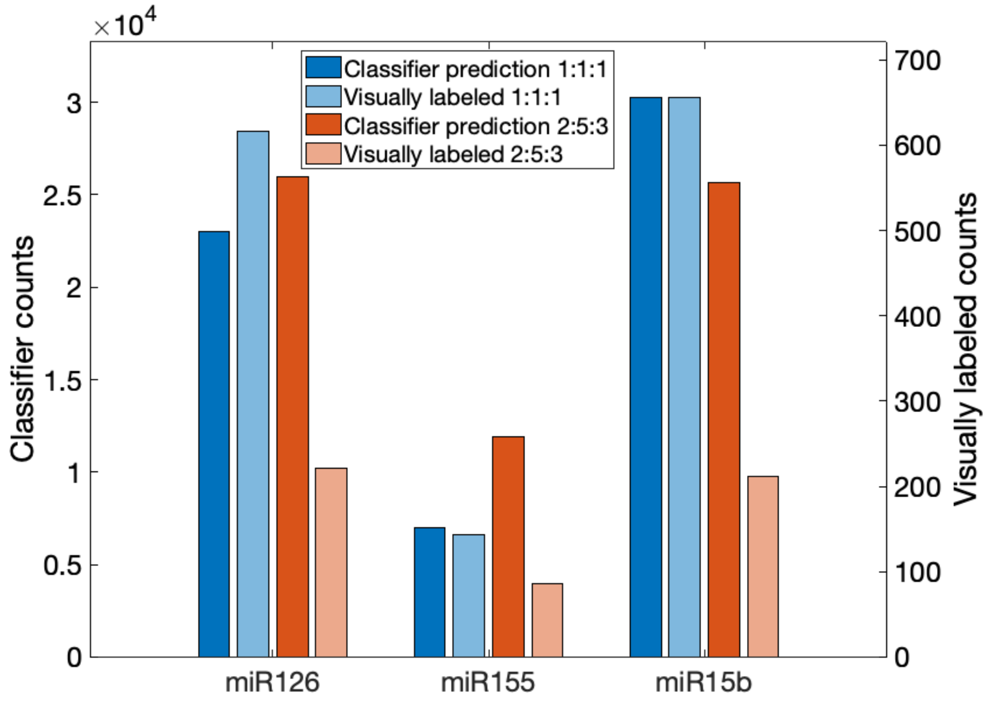

Figure S10 - Mixtures' absolute counts distributions. The left y-axis represents the total number of counts produced by the classifier whereas the right y-axis represents the number of crops visually classified by users using V-TIMDER.

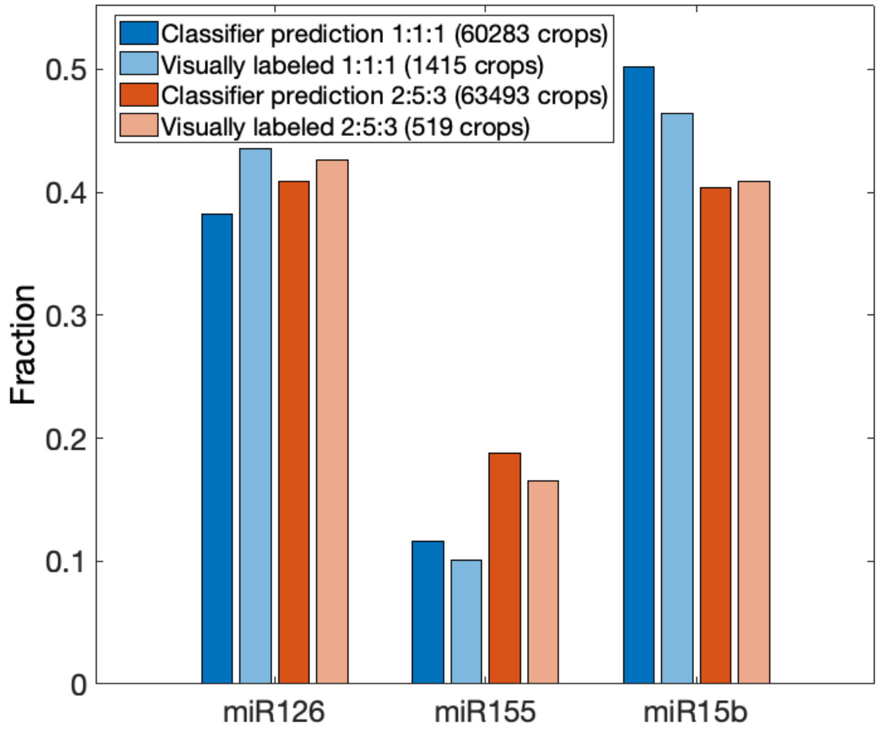

Figure S11 - Mixtures' fraction distributions. The total number of classified crops for each distribution is given in the legend.

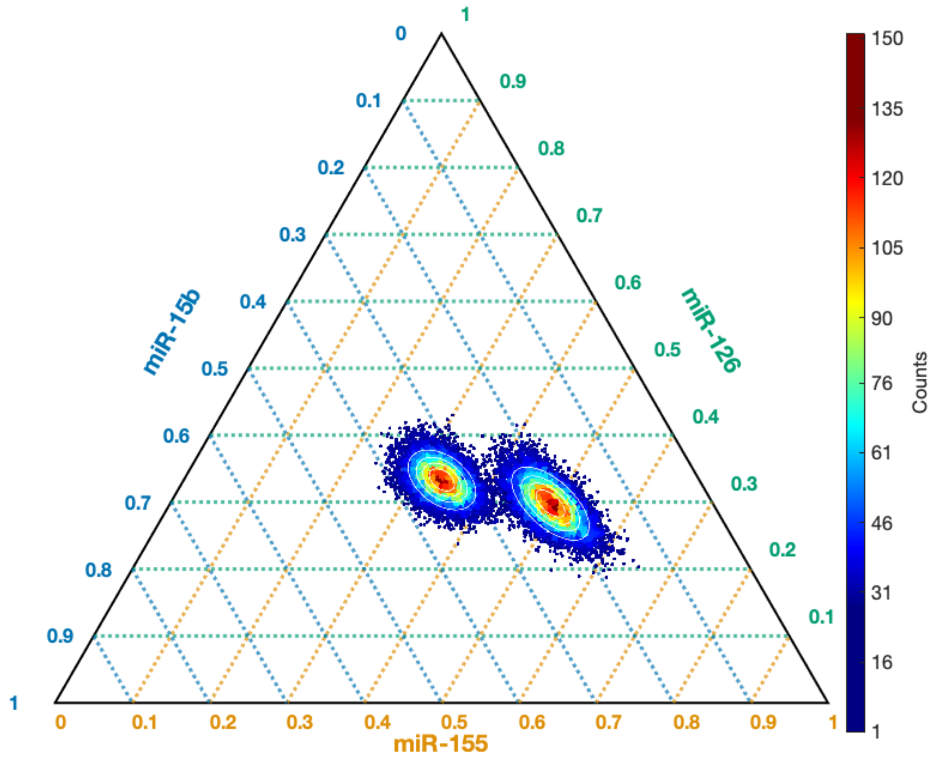

Figure S12 - MiR mixture distributions visualization on the concentrations 2-simplex by a ternary plot of miR concentration distributions for the two experimental mixtures 1:1:1 (0.33:0.33:0.33) and 2:5:3 (0.2:0.5:0.3). Color represents the binned counts of simulated concentration values according to the multinomial and Gaussian error estimation described in the methods. The white contour lines represent Gaussians fitted to each of the distributions. The plot was generated using "Ternary Plots" from Matlab's file exchange functions [2].

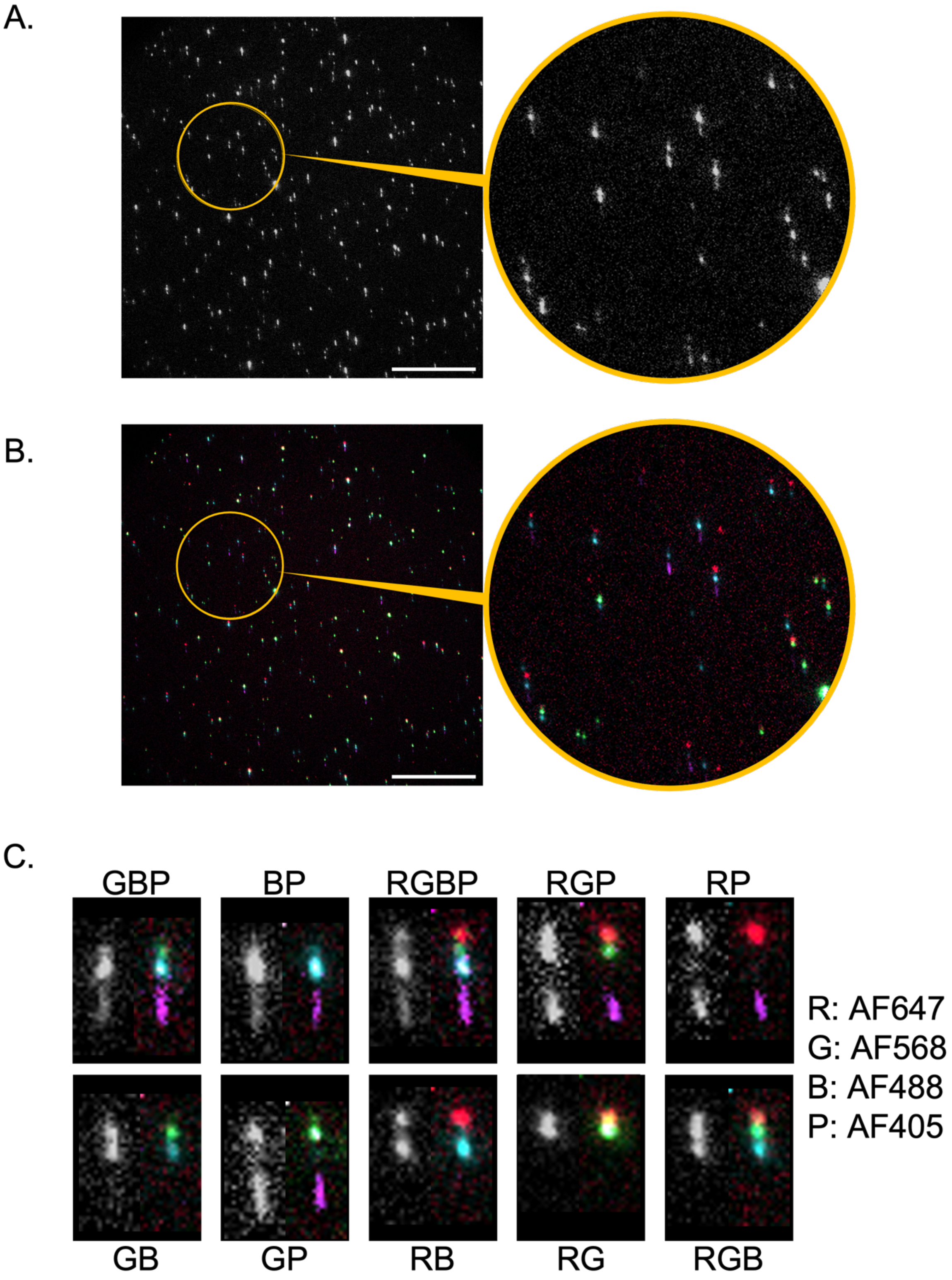

Figure S13 – Ten spectral PSF combinations and classification demonstrated by 100 nm silica beads labeled with four fluorophores. A) Spectral FOV of multi-color beads as registered by CoCoS with RPA=177° B) The same FOV sequentially excited by four different lasers (405 nm, 488 nm, 561 nm, and 638 nm), registered separately, false colored and overlayed to generate a four-color image. C) Example PSFs cropped and placed side-by-side showcasing the ten different PSFs according to their fluorophore combinations.

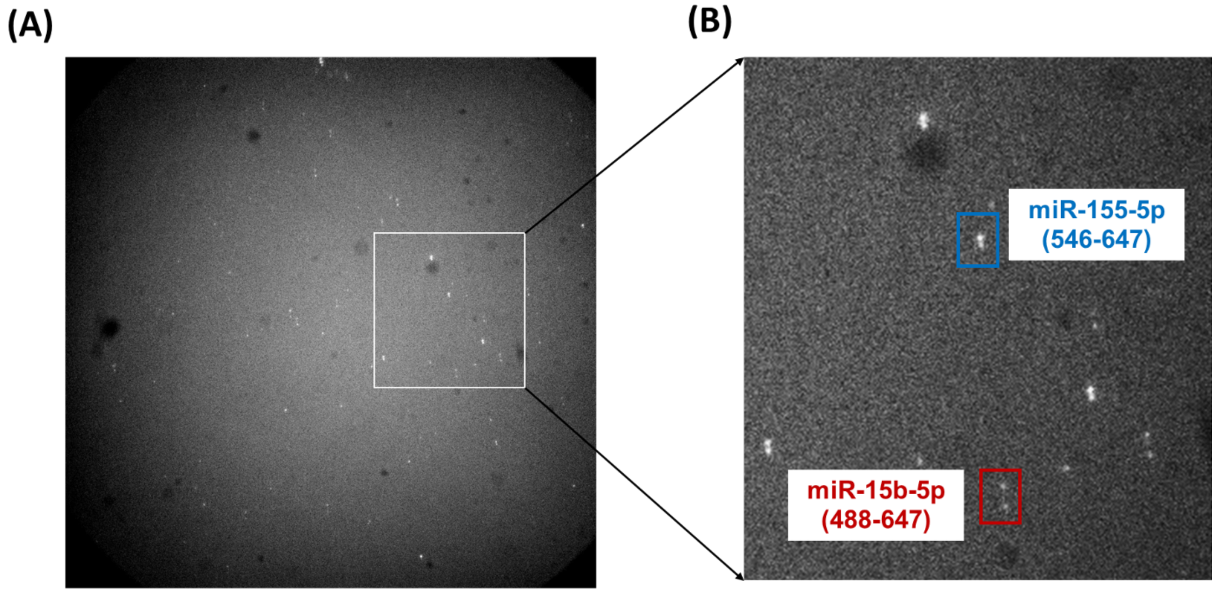

Figure S14 - Amplification-free detection of miR-15b-5p and miR-155 in small RNA extracted from 500 µL plasma. A) An example raw image of FOV containing the two miR targets. B) Zoom-in of the squared region in A, highlighting the two different PSFs, their corresponding miR targets identities and their fluorophore-pairs (in parentheses).

6. Crop size selection:

Crop dimensions, 24×10 pixels, were selected such that each crop will contain the complete point-spread-function (PSF) information of the three miR targets' spectral signatures. Therefore, the top blob of each TS detected PSF was centered around the sixth pixel from the top of the crop and at the 5th pixel from the left (centered), leaving 18 pixels below to encapsulate the bottom blob (maximal anticipated peaks distance of 11 pixels, see figure 1C in the main text). The empirical standard deviation of the blobs was determined as ~1.6 pixels, therefore the chosen dimensions of the crops allow it to contain the complete information of a single PSF, while minimizing the chance for detection of multiple PSFs within the same crop.

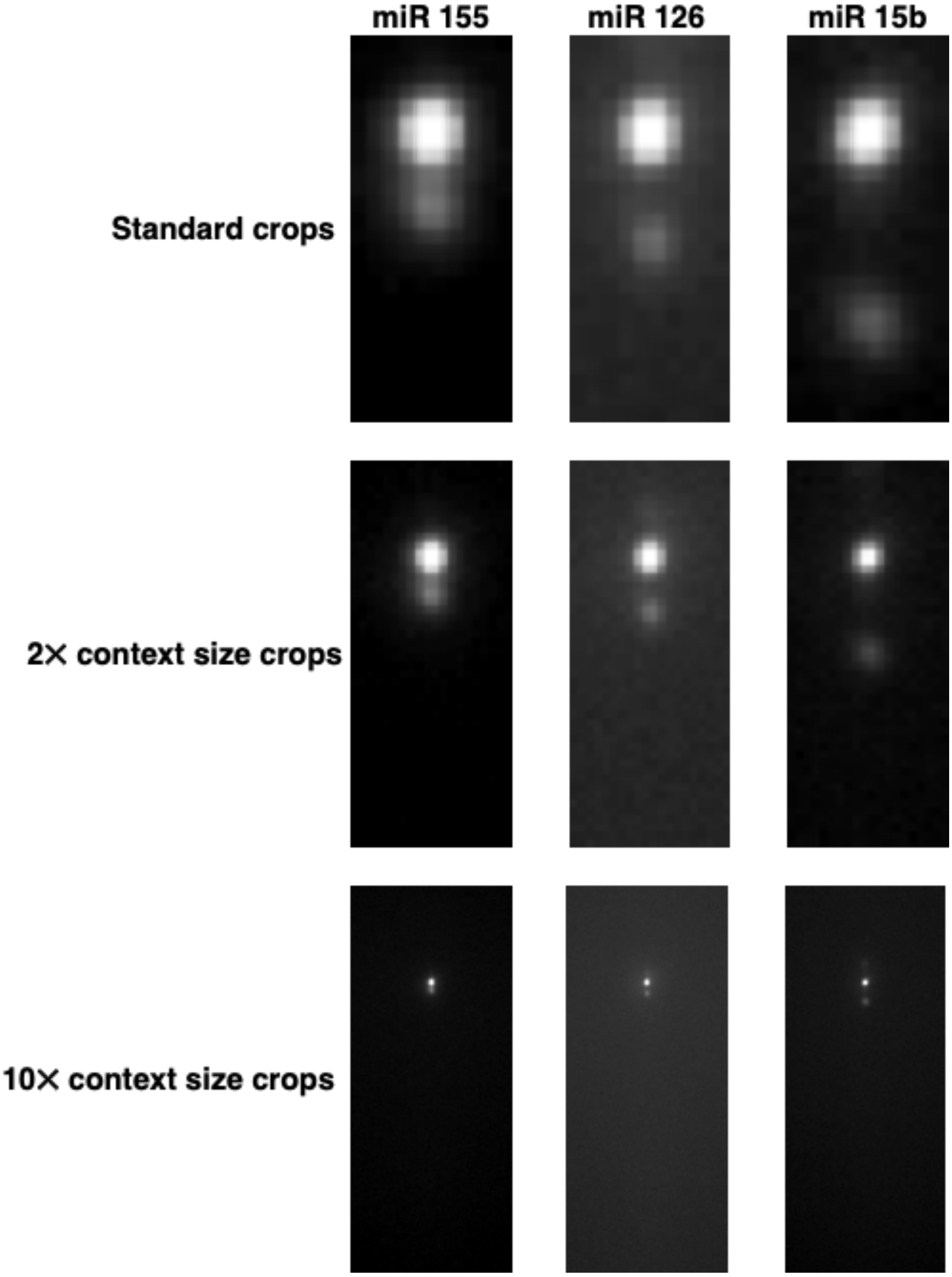

Figure S15 - Empirical PSF estimation crops. Empirical reference crops calculated from pixel-wise median of ~10^5 noisy crops. These reference images were input to V-TIMDER (see figure S6) to facilitate the PSF visual classification process.

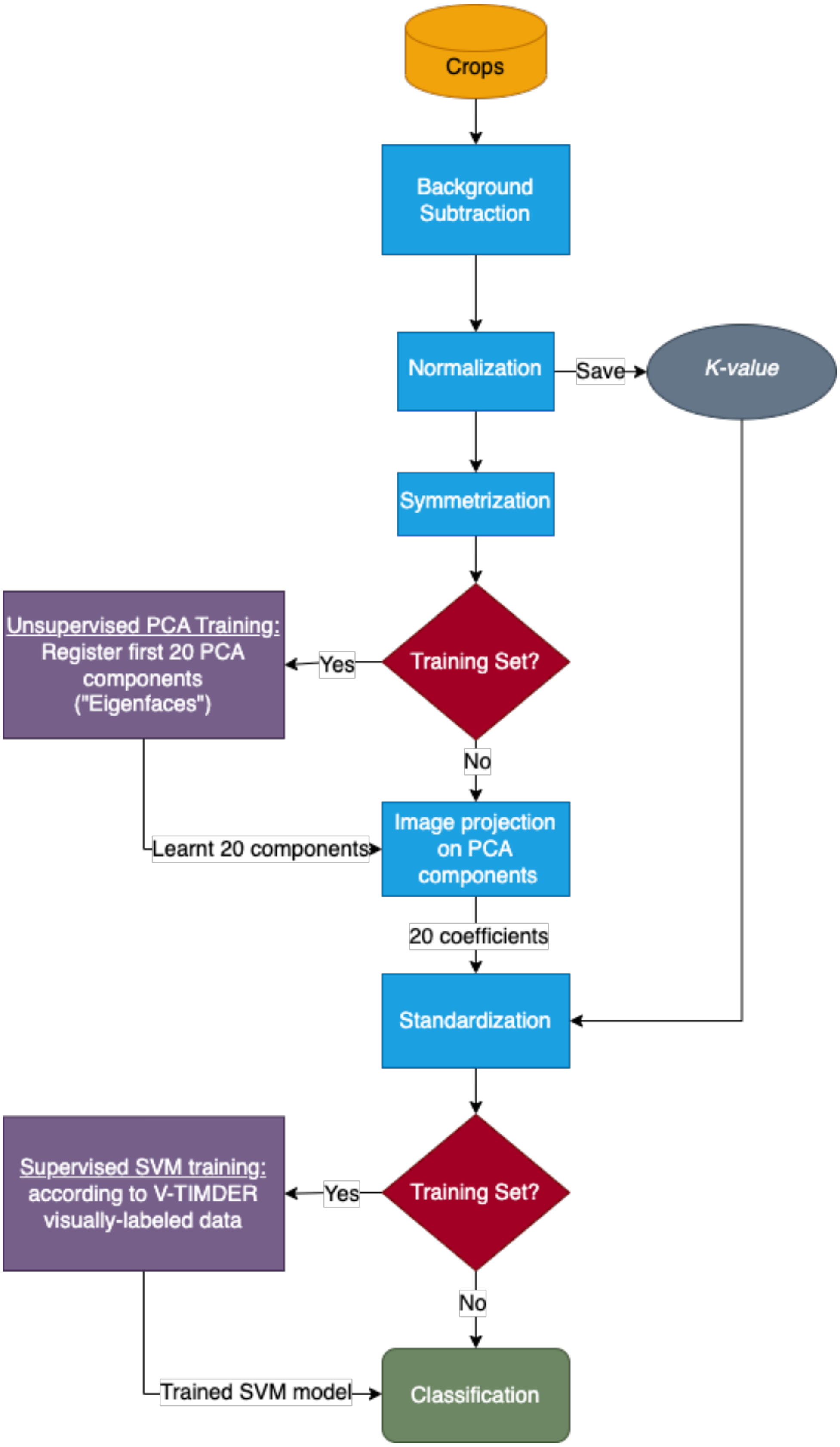

Figure S16 - A schematic diagram of the classifier's pipeline.

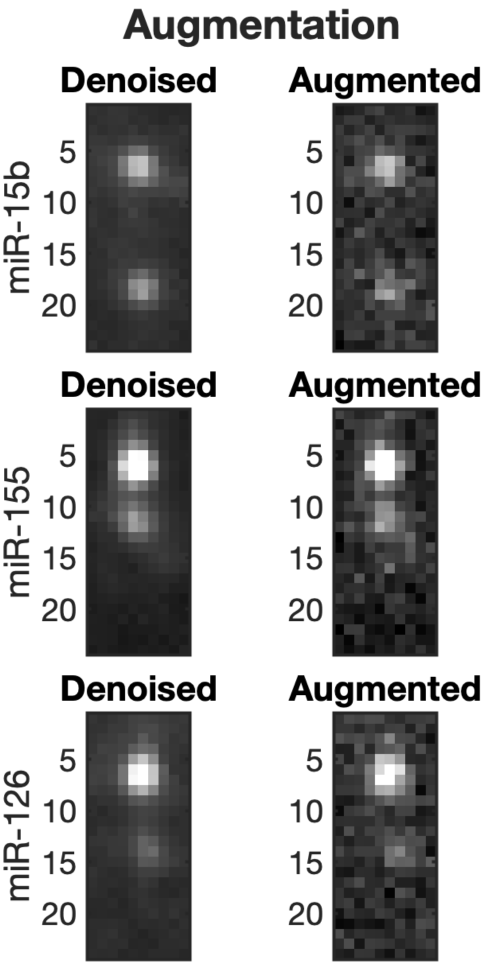

Figure S17 - Crop augmentation by addition of weak Gaussian noise according to table S5 parameters. Left column, three examples of the denoised miR crops. Right column, the same crops as the left column but with the addition of Gaussian noise. For each crop, six realizations of Gaussian augmented crops were made (see methods).

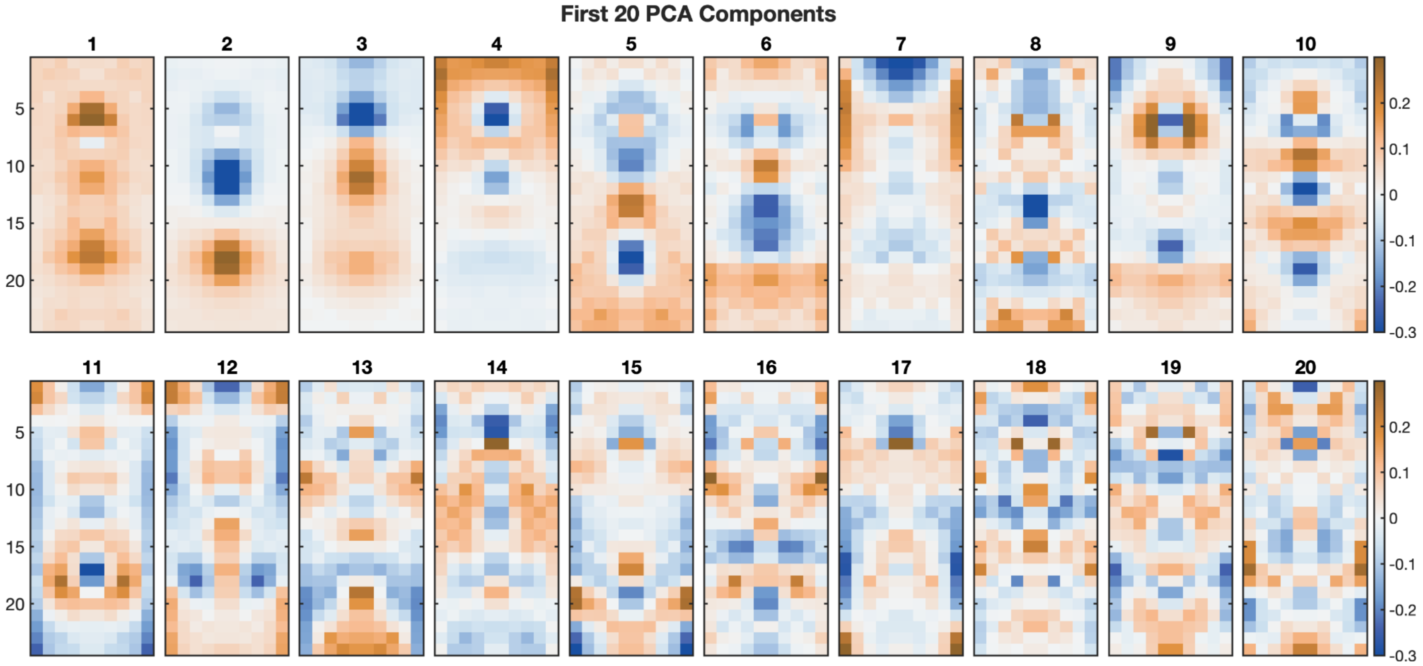

Figure S18 - The first 20 PCA components generated by the unsupervised PCA analysis. For visual clarity, the components were symmetrically mirrored to generate 24x10 pixels crops (instead of the actual 24x5 pixels generated by the PCA after the symmetrization preprocessing in the classifier's pipeline, see methods). The three spectral PSFs (5,7- and 11-pixel distances) are clearly represented in the first 6 components, whereas the rest are probably attributed to noise classification. The diverging colormap used to present the PCA components was generated using the 'lbmap' function downloaded from Matlab file exchange [3].

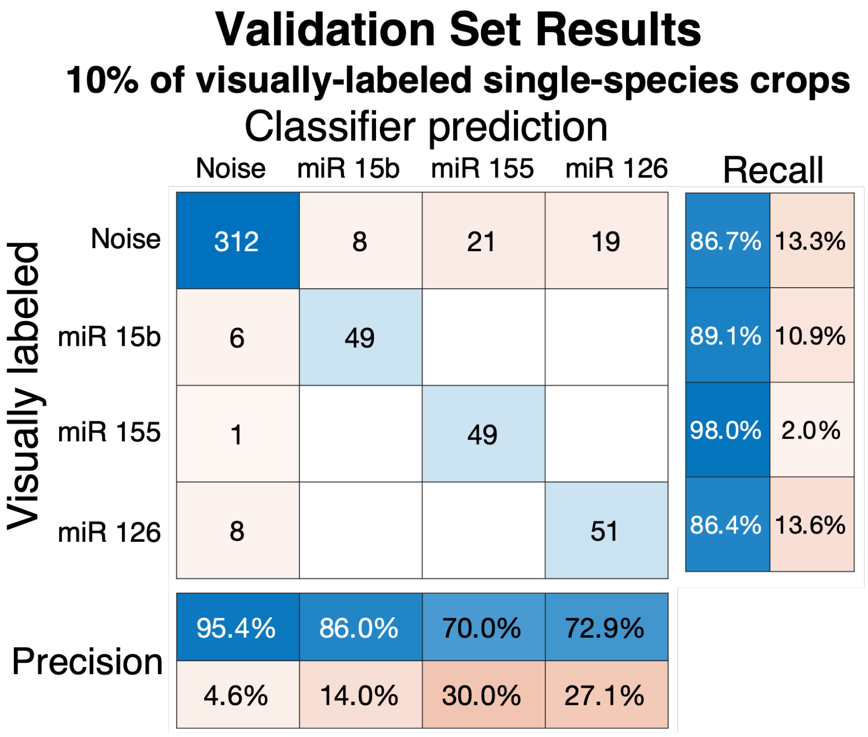

Figure S19 - Confusion matrix results for the validation set.

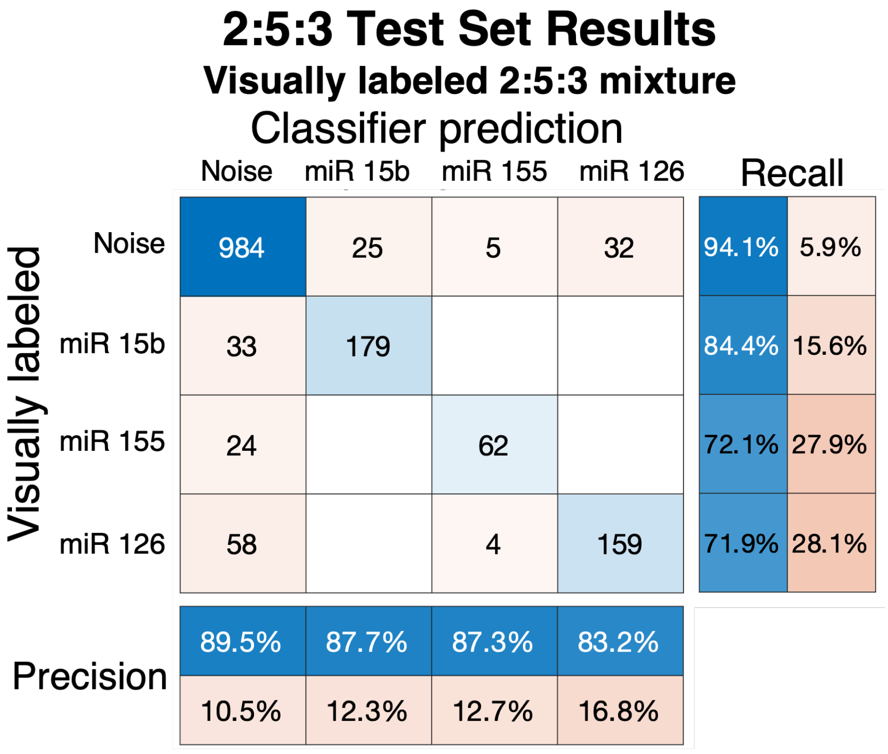

Figure S20 - Confusion matrix results for the visually labeled 2:5:3 mixture dataset.

7. <u>Protocol for glass coverslips cleaning and PEGylation</u>

Coverslips cleaning:

1. Mark coverslips on the right top side using diamond cutter tool. Take care to avoid glass breaking.
2. Rinse staining jar twice with isopropanol followed by ddH_2O.
3. Place marked coverslips in staining jar and rinse twice with isopropanol followed by ddH_2O.
4. Fill the staining jar with 2% Hellmanex. Sonicate 30 min at 30^0C and discard the solution.
5. Rinse the coverslips thoroughly with ddH_2O, repeat 8 times.
6. Sonicate the staining jar with ddH_2O for 10 minutes at 30^0C, and discard.
7. Fill the staining jar with freshly prepared 4M KOH (28.05g KOH in 125ml ddH_2O) and sonicate the slides for 100 min.
8. Rinse the coverslips thoroughly with ddH_2O, repeat 8 times to remove traces of KOH.
9. Feel the staining jar with technical grade (96%) ethanol, and discard.
10. Rinse the coverslips thoroughly with ddH_2O.
11. Blow dry the coverslips with nitrogen.
12. Bake the coverslips for 3 hours at 500°C (can be left overnight).
13. Rinse staining jar twice with ethanol followed by ddH_2O.
14. Place the coverslips in the cleaned staining jar, fill it with 1M KOH (7.01 g in 125ml ddH_2O) and sonicate 30 minutes at 30^0C.
15. Rinse the coverslips thoroughly with ddH_2O, repeat 8 times to remove all traces of KOH.

Coverslips PEGylation:

<u>Important note</u> – the lifetime of the PEG solution is short. Therefore, it must be prepared right before its application on coverslips.

1. Prepare PEG solution (3% by volume) in dehydrated high-grade ethanol (HPLC grade). For 500 µL of a 1:100 PEG-Biotin/mPEG Silane (for 4 coverslips):
   i. Dissolve 15 mg Methoxy PEG-Silane (mPEG-Silane MW5000, Laysan Bio Inc. AL, USA) and 0.15 mg Biotin-PEG-Silane (Biotin-PEG-Silane MW5000, Laysan Bio Inc. AL, USA) in 475 µL dehydrated ethanol. Mix by warming the solution for 1 minute to 35 °C followed by pipetting, **do not vortex**.
   ii. Add 25 µL glacial acetic acid (5% V/V) just before applying onto the coverslips.
2. Degas the solution: centrifuge for 1 min at 16,000g to remove air bubbles.
3. Blow dry the pairs of cleaned coverslips with nitrogen and place in an empty pipette tips box partially filled with ddH_2O.
4. For each pair of coverslips, sandwich 250 µL of the PEG solution between the coverslips (the engraved side facing to the center), avoid creating bubbles.
5. Cover box with tin foil and incubate overnight in a dark place at room temperature.
6. Carefully separate the coverslip pairs, take care not to break the glass.
7. Rinse carefully with ethanol followed by ddH_2O and thoroughly blow dry with nitrogen.
8. Store each coverslip separately in a 50 ml falcon filled with nitrogen. Seal the cap with parafilm and store in -20 °C.

References:

(1) Jeffet, J.; Ionescu, A.; Michaeli, Y.; Torchinsky, D.; Perlson, E.; Craggs, T. D.; Ebenstein, Y. Multimodal Single-Molecule Microscopy with Continuously Controlled Spectral Resolution. *Biophysical Reports* **2021**, *1* (1), 100013. https://doi.org/10.1016/j.bpr.2021.100013.
(2) Ulrich Theune. *Ternary Plots*. MATLAB Central File Exchange. https://www.mathworks.com/matlabcentral/fileexchange/7210-ternary-plots (accessed 2023-06-01).
(3) Robert Bemis. *Light Bartlein Color Maps*. MATLAB Central File Exchange. https://www.mathworks.com/matlabcentral/fileexchange/17555-light-bartlein-color-maps (accessed 2023-06-01).
